# Search for charged Higgs bosons produced in vector boson fusion processes and decaying into vector boson pairs in proton–proton collisions at $\sqrt{s} = 13\,{\text {TeV}}$

**DOI:** 10.1140/epjc/s10052-021-09472-3

**Published:** 2021-08-11

**Authors:** A. M. Sirunyan, A. Tumasyan, W. Adam, J. W. Andrejkovic, T. Bergauer, S. Chatterjee, M. Dragicevic, A. Escalante Del Valle, R. Frühwirth, M. Jeitler, N. Krammer, L. Lechner, D. Liko, I. Mikulec, P. Paulitsch, F. M. Pitters, J. Schieck, R. Schöfbeck, M. Spanring, S. Templ, W. Waltenberger, C.-E. Wulz, V. Chekhovsky, A. Litomin, V. Makarenko, M. R. Darwish, E. A. De Wolf, X. Janssen, T. Kello, A. Lelek, H. Rejeb Sfar, P. Van Mechelen, S. Van Putte, N. Van Remortel, F. Blekman, E. S. Bols, J. D’Hondt, J. De Clercq, M. Delcourt, H. El Faham, S. Lowette, S. Moortgat, A. Morton, D. Müller, A. R. Sahasransu, S. Tavernier, W. Van Doninck, P. Van Mulders, D. Beghin, B. Bilin, B. Clerbaux, G. De Lentdecker, L. Favart, A. Grebenyuk, A. K. Kalsi, K. Lee, M. Mahdavikhorrami, I. Makarenko, L. Moureaux, L. Pétré, A. Popov, N. Postiau, E. Starling, L. Thomas, M. Vanden Bemden, C. Vander Velde, P. Vanlaer, D. Vannerom, L. Wezenbeek, T. Cornelis, D. Dobur, J. Knolle, L. Lambrecht, G. Mestdach, M. Niedziela, C. Roskas, A. Samalan, K. Skovpen, M. Tytgat, W. Verbeke, B. Vermassen, M. Vit, A. Bethani, G. Bruno, F. Bury, C. Caputo, P. David, C. Delaere, I. S. Donertas, A. Giammanco, K. Jaffel, Sa. Jain, V. Lemaitre, K. Mondal, J. Prisciandaro, A. Taliercio, M. Teklishyn, T. T. Tran, P. Vischia, S. Wertz, S. Wuyckens, G. A. Alves, C. Hensel, A. Moraes, W. L. Aldá Júnior, M. Alves Gallo Pereira, M. Barroso Ferreira Filho, H. Brandao Malbouisson, W. Carvalho, J. Chinellato, E. M. Da Costa, G. G. Da Silveira, D. De Jesus Damiao, S. Fonseca De Souza, D. Matos Figueiredo, C. Mora Herrera, K. Mota Amarilo, L. Mundim, H. Nogima, P. Rebello Teles, A. Santoro, S. M. Silva Do Amaral, A. Sznajder, M. Thiel, F. Torres Da Silva De Araujo, A. Vilela Pereira, C. A. Bernardes, L. Calligaris, T. R. Fernandez Perez Tomei, E. M. Gregores, D. S. Lemos, P. G. Mercadante, S. F. Novaes, Sandra S. Padula, A. Aleksandrov, G. Antchev, R. Hadjiiska, P. Iaydjiev, M. Misheva, M. Rodozov, M. Shopova, G. Sultanov, A. Dimitrov, T. Ivanov, L. Litov, B. Pavlov, P. Petkov, A. Petrov, T. Cheng, W. Fang, Q. Guo, T. Javaid, M. Mittal, H. Wang, L. Yuan, M. Ahmad, G. Bauer, C. Dozen, Z. Hu, J. Martins, Y. Wang, K. Yi, E. Chapon, G. M. Chen, H. S. Chen, M. Chen, F. Iemmi, A. Kapoor, D. Leggat, H. Liao, Z.-A. Liu, V. Milosevic, F. Monti, R. Sharma, J. Tao, J. Thomas-wilsker, J. Wang, H. Zhang, S. Zhang, J. Zhao, A. Agapitos, Y. Ban, C. Chen, Q. Huang, A. Levin, Q. Li, X. Lyu, Y. Mao, S. J. Qian, D. Wang, Q. Wang, J. Xiao, M. Lu, Z. You, X. Gao, H. Okawa, Z. Lin, M. Xiao, C. Avila, A. Cabrera, C. Florez, J. Fraga, A. Sarkar, M. A. Segura Delgado, J. Mejia Guisao, F. Ramirez, J. D. Ruiz Alvarez, C. A. Salazar González, D. Giljanovic, N. Godinovic, D. Lelas, I. Puljak, Z. Antunovic, M. Kovac, T. Sculac, V. Brigljevic, D. Ferencek, D. Majumder, M. Roguljic, A. Starodumov, T. Susa, A. Attikis, K. Christoforou, E. Erodotou, A. Ioannou, G. Kole, M. Kolosova, S. Konstantinou, J. Mousa, C. Nicolaou, F. Ptochos, P. A. Razis, H. Rykaczewski, H. Saka, M. Finger, M. Finger, A. Kveton, E. Ayala, E. Carrera Jarrin, H. Abdalla, A. A. Abdelalim, A. Lotfy, M. A. Mahmoud, S. Bhowmik, A. Carvalho Antunes De Oliveira, R. K. Dewanjee, K. Ehataht, M. Kadastik, S. Nandan, C. Nielsen, J. Pata, M. Raidal, L. Tani, C. Veelken, P. Eerola, L. Forthomme, H. Kirschenmann, K. Osterberg, M. Voutilainen, S. Bharthuar, E. Brücken, F. Garcia, J. Havukainen, M. S. Kim, R. Kinnunen, T. Lampén, K. Lassila-Perini, S. Lehti, T. Lindén, M. Lotti, L. Martikainen, M. Myllymäki, J. Ott, H. Siikonen, E. Tuominen, J. Tuominiemi, P. Luukka, H. Petrow, T. Tuuva, C. Amendola, M. Besancon, F. Couderc, M. Dejardin, D. Denegri, J. L. Faure, F. Ferri, S. Ganjour, A. Givernaud, P. Gras, G. Hamel de Monchenault, P. Jarry, B. Lenzi, E. Locci, J. Malcles, J. Rander, A. Rosowsky, M. Ö. Sahin, A. Savoy-Navarro, M. Titov, G. B. Yu, S. Ahuja, F. Beaudette, M. Bonanomi, A. Buchot Perraguin, P. Busson, A. Cappati, C. Charlot, O. Davignon, B. Diab, G. Falmagne, S. Ghosh, R. Granier de Cassagnac, A. Hakimi, I. Kucher, M. Nguyen, C. Ochando, P. Paganini, J. Rembser, R. Salerno, J. B. Sauvan, Y. Sirois, A. Zabi, A. Zghiche, J.-L. Agram, J. Andrea, D. Apparu, D. Bloch, G. Bourgatte, J.-M. Brom, E. C. Chabert, C. Collard, D. Darej, J.-C. Fontaine, U. Goerlach, C. Grimault, A.-C. Le Bihan, E. Nibigira, P. Van Hove, E. Asilar, S. Beauceron, C. Bernet, G. Boudoul, C. Camen, A. Carle, N. Chanon, D. Contardo, P. Depasse, H. El Mamouni, J. Fay, S. Gascon, M. Gouzevitch, B. Ille, I. B. Laktineh, H. Lattaud, A. Lesauvage, M. Lethuillier, L. Mirabito, S. Perries, K. Shchablo, V. Sordini, L. Torterotot, G. Touquet, M. Vander Donckt, S. Viret, A. Khvedelidze, I. Lomidze, Z. Tsamalaidze, L. Feld, K. Klein, M. Lipinski, D. Meuser, A. Pauls, M. P. Rauch, N. Röwert, J. Schulz, M. Teroerde, A. Dodonova, D. Eliseev, M. Erdmann, P. Fackeldey, B. Fischer, S. Ghosh, T. Hebbeker, K. Hoepfner, F. Ivone, H. Keller, L. Mastrolorenzo, M. Merschmeyer, A. Meyer, G. Mocellin, S. Mondal, S. Mukherjee, D. Noll, A. Novak, T. Pook, A. Pozdnyakov, Y. Rath, H. Reithler, J. Roemer, A. Schmidt, S. C. Schuler, A. Sharma, L. Vigilante, S. Wiedenbeck, S. Zaleski, C. Dziwok, G. Flügge, W. Haj Ahmad, O. Hlushchenko, T. Kress, A. Nowack, C. Pistone, O. Pooth, D. Roy, H. Sert, A. Stahl, T. Ziemons, H. Aarup Petersen, M. Aldaya Martin, P. Asmuss, I. Babounikau, S. Baxter, O. Behnke, A. Bermúdez Martínez, S. Bhattacharya, A. A. Bin Anuar, K. Borras, V. Botta, D. Brunner, A. Campbell, A. Cardini, C. Cheng, F. Colombina, S. Consuegra Rodríguez, G. Correia Silva, V. Danilov, L. Didukh, G. Eckerlin, D. Eckstein, L. I. Estevez Banos, O. Filatov, E. Gallo, A. Geiser, A. Giraldi, A. Grohsjean, M. Guthoff, A. Jafari, N. Z. Jomhari, H. Jung, A. Kasem, M. Kasemann, H. Kaveh, C. Kleinwort, D. Krücker, W. Lange, J. Lidrych, K. Lipka, W. Lohmann, R. Mankel, I.-A. Melzer-Pellmann, J. Metwally, A. B. Meyer, M. Meyer, J. Mnich, A. Mussgiller, Y. Otarid, D. Pérez Adán, D. Pitzl, A. Raspereza, B. Ribeiro Lopes, J. Rübenach, A. Saggio, A. Saibel, M. Savitskyi, M. Scham, V. Scheurer, C. Schwanenberger, A. Singh, R. E. Sosa Ricardo, D. Stafford, N. Tonon, O. Turkot, M. Van De Klundert, R. Walsh, D. Walter, Y. Wen, K. Wichmann, L. Wiens, C. Wissing, S. Wuchterl, R. Aggleton, S. Albrecht, S. Bein, L. Benato, A. Benecke, P. Connor, K. De Leo, M. Eich, F. Feindt, A. Fröhlich, C. Garbers, E. Garutti, P. Gunnellini, J. Haller, A. Hinzmann, G. Kasieczka, R. Klanner, R. Kogler, T. Kramer, V. Kutzner, J. Lange, T. Lange, A. Lobanov, A. Malara, A. Nigamova, K. J. Pena Rodriguez, O. Rieger, P. Schleper, M. Schröder, J. Schwandt, D. Schwarz, J. Sonneveld, H. Stadie, G. Steinbrück, A. Tews, B. Vormwald, I. Zoi, J. Bechtel, T. Berger, E. Butz, R. Caspart, T. Chwalek, W. De Boer, A. Dierlamm, A. Droll, K. El Morabit, N. Faltermann, M. Giffels, J. O. Gosewisch, A. Gottmann, F. Hartmann, C. Heidecker, U. Husemann, I. Katkov, P. Keicher, R. Koppenhöfer, S. Maier, M. Metzler, S. Mitra, Th. Müller, M. Neukum, A. Nürnberg, G. Quast, K. Rabbertz, J. Rauser, D. Savoiu, M. Schnepf, D. Seith, I. Shvetsov, H. J. Simonis, R. Ulrich, J. Van Der Linden, R. F. Von Cube, M. Wassmer, M. Weber, S. Wieland, R. Wolf, S. Wozniewski, S. Wunsch, G. Anagnostou, P. Asenov, G. Daskalakis, T. Geralis, A. Kyriakis, D. Loukas, A. Stakia, M. Diamantopoulou, D. Karasavvas, G. Karathanasis, P. Kontaxakis, C. K. Koraka, A. Manousakis-katsikakis, A. Panagiotou, I. Papavergou, N. Saoulidou, K. Theofilatos, E. Tziaferi, K. Vellidis, E. Vourliotis, G. Bakas, K. Kousouris, I. Papakrivopoulos, G. Tsipolitis, A. Zacharopoulou, I. Evangelou, C. Foudas, P. Gianneios, P. Katsoulis, P. Kokkas, N. Manthos, I. Papadopoulos, J. Strologas, M. Csanad, K. Farkas, M. M. A. Gadallah, S. Lökös, P. Major, K. Mandal, A. Mehta, G. Pasztor, A. J. Rádl, O. Surányi, G. I. Veres, M. Bartók, G. Bencze, C. Hajdu, D. Horvath, F. Sikler, V. Veszpremi, G. Vesztergombi, S. Czellar, J. Karancsi, J. Molnar, Z. Szillasi, D. Teyssier, P. Raics, Z. L. Trocsanyi, B. Ujvari, T. Csorgo, F. Nemes, T. Novak, J. R. Komaragiri, D. Kumar, L. Panwar, P. C. Tiwari, S. Bahinipati, D. Dash, C. Kar, P. Mal, T. Mishra, V. K. Muraleedharan Nair Bindhu, A. Nayak, P. Saha, N. Sur, S. K. Swain, D. Vats, S. Bansal, S. B. Beri, V. Bhatnagar, G. Chaudhary, S. Chauhan, N. Dhingra, R. Gupta, A. Kaur, M. Kaur, S. Kaur, P. Kumari, M. Meena, K. Sandeep, J. B. Singh, A. K. Virdi, A. Ahmed, A. Bhardwaj, B. C. Choudhary, M. Gola, S. Keshri, A. Kumar, M. Naimuddin, P. Priyanka, K. Ranjan, A. Shah, M. Bharti, R. Bhattacharya, S. Bhattacharya, D. Bhowmik, S. Dutta, S. Dutta, B. Gomber, M. Maity, P. Palit, P. K. Rout, G. Saha, B. Sahu, S. Sarkar, M. Sharan, B. Singh, S. Thakur, P. K. Behera, S. C. Behera, P. Kalbhor, A. Muhammad, R. Pradhan, P. R. Pujahari, A. Sharma, A. K. Sikdar, D. Dutta, V. Jha, V. Kumar, D. K. Mishra, K. Naskar, P. K. Netrakanti, L. M. Pant, P. Shukla, T. Aziz, S. Dugad, M. Kumar, U. Sarkar, S. Banerjee, R. Chudasama, M. Guchait, S. Karmakar, S. Kumar, G. Majumder, K. Mazumdar, S. Mukherjee, K. Alpana, S. Dube, B. Kansal, A. Laha, S. Pandey, A. Rane, A. Rastogi, S. Sharma, H. Bakhshiansohi, M. Zeinali, S. Chenarani, S. M. Etesami, M. Khakzad, M. Mohammadi Najafabadi, M. Grunewald, M. Abbrescia, R. Aly, C. Aruta, A. Colaleo, D. Creanza, N. De Filippis, M. De Palma, A. Di Florio, A. Di Pilato, W. Elmetenawee, L. Fiore, A. Gelmi, M. Gul, G. Iaselli, M. Ince, S. Lezki, G. Maggi, M. Maggi, I. Margjeka, V. Mastrapasqua, J. A. Merlin, S. My, S. Nuzzo, A. Pellecchia, A. Pompili, G. Pugliese, A. Ranieri, G. Selvaggi, L. Silvestris, F. M. Simone, R. Venditti, P. Verwilligen, G. Abbiendi, C. Battilana, D. Bonacorsi, L. Borgonovi, L. Brigliadori, R. Campanini, P. Capiluppi, A. Castro, F. R. Cavallo, M. Cuffiani, G. M. Dallavalle, T. Diotalevi, F. Fabbri, A. Fanfani, P. Giacomelli, L. Giommi, C. Grandi, L. Guiducci, S. Lo Meo, L. Lunerti, S. Marcellini, G. Masetti, F. L. Navarria, A. Perrotta, F. Primavera, A. M. Rossi, T. Rovelli, G. P. Siroli, S. Albergo, S. Costa, A. Di Mattia, R. Potenza, A. Tricomi, C. Tuve, G. Barbagli, A. Cassese, R. Ceccarelli, V. Ciulli, C. Civinini, R. D’Alessandro, E. Focardi, G. Latino, P. Lenzi, M. Lizzo, M. Meschini, S. Paoletti, R. Seidita, G. Sguazzoni, L. Viliani, L. Benussi, S. Bianco, D. Piccolo, M. Bozzo, F. Ferro, R. Mulargia, E. Robutti, S. Tosi, A. Benaglia, F. Brivio, F. Cetorelli, V. Ciriolo, F. De Guio, M. E. Dinardo, P. Dini, S. Gennai, A. Ghezzi, P. Govoni, L. Guzzi, M. Malberti, S. Malvezzi, A. Massironi, D. Menasce, L. Moroni, M. Paganoni, D. Pedrini, S. Ragazzi, N. Redaelli, T. Tabarelli de Fatis, D. Valsecchi, D. Zuolo, S. Buontempo, F. Carnevali, N. Cavallo, A. De Iorio, F. Fabozzi, A. O. M. Iorio, L. Lista, S. Meola, P. Paolucci, B. Rossi, C. Sciacca, P. Azzi, N. Bacchetta, D. Bisello, P. Bortignon, A. Bragagnolo, R. Carlin, P. Checchia, T. Dorigo, U. Dosselli, F. Gasparini, U. Gasparini, S. Y. Hoh, L. Layer, M. Margoni, A. T. Meneguzzo, J. Pazzini, M. Presilla, P. Ronchese, R. Rossin, F. Simonetto, G. Strong, M. Tosi, H. Yarar, M. Zanetti, P. Zotto, A. Zucchetta, G. Zumerle, C. Aimé, A. Braghieri, S. Calzaferri, D. Fiorina, P. Montagna, S. P. Ratti, V. Re, C. Riccardi, P. Salvini, I. Vai, P. Vitulo, G. M. Bilei, D. Ciangottini, L. Fanò, P. Lariccia, M. Magherini, G. Mantovani, V. Mariani, M. Menichelli, F. Moscatelli, A. Piccinelli, A. Rossi, A. Santocchia, D. Spiga, T. Tedeschi, P. Azzurri, G. Bagliesi, V. Bertacchi, L. Bianchini, T. Boccali, E. Bossini, R. Castaldi, M. A. Ciocci, V. D’Amante, R. Dell’Orso, M. R. Di Domenico, S. Donato, A. Giassi, M. T. Grippo, F. Ligabue, E. Manca, G. Mandorli, A. Messineo, F. Palla, S. Parolia, G. Ramirez-Sanchez, A. Rizzi, G. Rolandi, S. Roy Chowdhury, A. Scribano, N. Shafiei, P. Spagnolo, R. Tenchini, G. Tonelli, N. Turini, A. Venturi, P. G. Verdini, M. Campana, F. Cavallari, M. Cipriani, D. Del Re, E. Di Marco, M. Diemoz, E. Longo, P. Meridiani, G. Organtini, F. Pandolfi, R. Paramatti, C. Quaranta, S. Rahatlou, C. Rovelli, F. Santanastasio, L. Soffi, R. Tramontano, N. Amapane, R. Arcidiacono, S. Argiro, M. Arneodo, N. Bartosik, R. Bellan, A. Bellora, J. Berenguer Antequera, C. Biino, N. Cartiglia, S. Cometti, M. Costa, R. Covarelli, N. Demaria, B. Kiani, F. Legger, C. Mariotti, S. Maselli, E. Migliore, E. Monteil, M. Monteno, M. M. Obertino, G. Ortona, L. Pacher, N. Pastrone, M. Pelliccioni, G. L. Pinna Angioni, M. Ruspa, K. Shchelina, F. Siviero, V. Sola, A. Solano, D. Soldi, A. Staiano, M. Tornago, D. Trocino, A. Vagnerini, S. Belforte, V. Candelise, M. Casarsa, F. Cossutti, A. Da Rold, G. Della Ricca, G. Sorrentino, F. Vazzoler, S. Dogra, C. Huh, B. Kim, D. H. Kim, G. N. Kim, J. Kim, J. Lee, S. W. Lee, C. S. Moon, Y. D. Oh, S. I. Pak, B. C. Radburn-Smith, S. Sekmen, Y. C. Yang, H. Kim, D. H. Moon, B. Francois, T. J. Kim, J. Park, S. Cho, S. Choi, Y. Go, B. Hong, K. Lee, K. S. Lee, J. Lim, J. Park, S. K. Park, J. Yoo, J. Goh, A. Gurtu, H. S. Kim, Y. Kim, J. Almond, J. H. Bhyun, J. Choi, S. Jeon, J. Kim, J. S. Kim, S. Ko, H. Kwon, H. Lee, S. Lee, B. H. Oh, M. Oh, S. B. Oh, H. Seo, U. K. Yang, I. Yoon, W. Jang, D. Jeon, D. Y. Kang, Y. Kang, J. H. Kim, S. Kim, B. Ko, J. S. H. Lee, Y. Lee, I. C. Park, Y. Roh, M. S. Ryu, D. Song, I. J. Watson, S. Yang, S. Ha, H. D. Yoo, M. Choi, Y. Jeong, H. Lee, Y. Lee, I. Yu, T. Beyrouthy, Y. Maghrbi, V. Veckalns, M. Ambrozas, A. Juodagalvis, A. Rinkevicius, G. Tamulaitis, N. Bin Norjoharuddeen, W. A. T. Wan Abdullah, M. N. Yusli, Z. Zolkapli, J. F. Benitez, A. Castaneda Hernandez, M. León Coello, J. A. Murillo Quijada, A. Sehrawat, L. Valencia Palomo, G. Ayala, H. Castilla-Valdez, E. De La Cruz-Burelo, I. Heredia-De La Cruz, R. Lopez-Fernandez, C. A. Mondragon Herrera, D. A. Perez Navarro, A. Sanchez-Hernandez, S. Carrillo Moreno, C. Oropeza Barrera, M. Ramirez-Garcia, F. Vazquez Valencia, I. Pedraza, H. A. Salazar Ibarguen, C. Uribe Estrada, J. Mijuskovic, N. Raicevic, D. Krofcheck, S. Bheesette, P. H. Butler, A. Ahmad, M. I. Asghar, A. Awais, M. I. M. Awan, H. R. Hoorani, W. A. Khan, M. A. Shah, M. Shoaib, M. Waqas, V. Avati, L. Grzanka, M. Malawski, H. Bialkowska, M. Bluj, B. Boimska, M. Górski, M. Kazana, M. Szleper, P. Zalewski, K. Bunkowski, K. Doroba, A. Kalinowski, M. Konecki, J. Krolikowski, M. Walczak, M. Araujo, P. Bargassa, D. Bastos, A. Boletti, P. Faccioli, M. Gallinaro, J. Hollar, N. Leonardo, T. Niknejad, M. Pisano, J. Seixas, O. Toldaiev, J. Varela, S. Afanasiev, D. Budkouski, I. Golutvin, I. Gorbunov, V. Karjavine, V. Korenkov, A. Lanev, A. Malakhov, V. Matveev, V. Palichik, V. Perelygin, M. Savina, D. Seitova, V. Shalaev, S. Shmatov, S. Shulha, V. Smirnov, O. Teryaev, N. Voytishin, B. S. Yuldashev, A. Zarubin, I. Zhizhin, G. Gavrilov, V. Golovtcov, Y. Ivanov, V. Kim, E. Kuznetsova, V. Murzin, V. Oreshkin, I. Smirnov, D. Sosnov, V. Sulimov, L. Uvarov, S. Volkov, A. Vorobyev, Yu. Andreev, A. Dermenev, S. Gninenko, N. Golubev, A. Karneyeu, D. Kirpichnikov, M. Kirsanov, N. Krasnikov, A. Pashenkov, G. Pivovarov, D. Tlisov, A. Toropin, V. Epshteyn, V. Gavrilov, N. Lychkovskaya, A. Nikitenko, V. Popov, A. Spiridonov, A. Stepennov, M. Toms, E. Vlasov, A. Zhokin, T. Aushev, M. Chadeeva, A. Oskin, P. Parygin, E. Popova, E. Zhemchugov, V. Andreev, M. Azarkin, I. Dremin, M. Kirakosyan, A. Terkulov, A. Belyaev, E. Boos, V. Bunichev, M. Dubinin, L. Dudko, A. Ershov, V. Klyukhin, O. Kodolova, I. Lokhtin, S. Obraztsov, M. Perfilov, S. Petrushanko, V. Savrin, V. Blinov, T. Dimova, L. Kardapoltsev, A. Kozyrev, I. Ovtin, Y. Skovpen, I. Azhgirey, I. Bayshev, D. Elumakhov, V. Kachanov, D. Konstantinov, P. Mandrik, V. Petrov, R. Ryutin, S. Slabospitskii, A. Sobol, S. Troshin, N. Tyurin, A. Uzunian, A. Volkov, A. Babaev, V. Okhotnikov, V. Borshch, V. Ivanchenko, E. Tcherniaev, P. Adzic, M. Dordevic, P. Milenovic, J. Milosevic, M. Aguilar-Benitez, J. Alcaraz Maestre, A. Álvarez Fernández, I. Bachiller, M. Barrio Luna, Cristina F. Bedoya, C. A. Carrillo Montoya, M. Cepeda, M. Cerrada, N. Colino, B. De La Cruz, A. Delgado Peris, J. P. Fernández Ramos, J. Flix, M. C. Fouz, O. Gonzalez Lopez, S. Goy Lopez, J. M. Hernandez, M. I. Josa, J. León Holgado, D. Moran, Á. Navarro Tobar, A. Pérez-Calero Yzquierdo, J. Puerta Pelayo, I. Redondo, L. Romero, S. Sánchez Navas, L. Urda Gómez, C. Willmott, J. F. de Trocóniz, R. Reyes-Almanza, B. Alvarez Gonzalez, J. Cuevas, C. Erice, J. Fernandez Menendez, S. Folgueras, I. Gonzalez Caballero, E. Palencia Cortezon, C. Ramón Álvarez, J. Ripoll Sau, V. Rodríguez Bouza, A. Trapote, N. Trevisani, J. A. Brochero Cifuentes, I. J. Cabrillo, A. Calderon, J. Duarte Campderros, M. Fernandez, C. Fernandez Madrazo, P. J. Fernández Manteca, A. García Alonso, G. Gomez, C. Martinez Rivero, P. Martinez Ruiz del Arbol, F. Matorras, P. Matorras Cuevas, J. Piedra Gomez, C. Prieels, T. Rodrigo, A. Ruiz-Jimeno, L. Scodellaro, I. Vila, J. M. Vizan Garcia, MK Jayananda, B. Kailasapathy, D. U. J. Sonnadara, DDC Wickramarathna, W. G. D. Dharmaratna, K. Liyanage, N. Perera, N. Wickramage, T. K. Aarrestad, D. Abbaneo, J. Alimena, E. Auffray, G. Auzinger, J. Baechler, P. Baillon, D. Barney, J. Bendavid, M. Bianco, A. Bocci, T. Camporesi, M. Capeans Garrido, G. Cerminara, S. S. Chhibra, L. Cristella, D. d’Enterria, A. Dabrowski, N. Daci, A. David, A. De Roeck, M. M. Defranchis, M. Deile, M. Dobson, M. Dünser, N. Dupont, A. Elliott-Peisert, N. Emriskova, F. Fallavollita, D. Fasanella, S. Fiorendi, A. Florent, G. Franzoni, W. Funk, S. Giani, D. Gigi, K. Gill, F. Glege, L. Gouskos, M. Haranko, J. Hegeman, Y. Iiyama, V. Innocente, T. James, P. Janot, J. Kaspar, J. Kieseler, M. Komm, N. Kratochwil, C. Lange, S. Laurila, P. Lecoq, K. Long, C. Lourenço, L. Malgeri, S. Mallios, M. Mannelli, A. C. Marini, F. Meijers, S. Mersi, E. Meschi, F. Moortgat, M. Mulders, S. Orfanelli, L. Orsini, F. Pantaleo, L. Pape, E. Perez, M. Peruzzi, A. Petrilli, G. Petrucciani, A. Pfeiffer, M. Pierini, D. Piparo, M. Pitt, H. Qu, T. Quast, D. Rabady, A. Racz, G. Reales Gutiérrez, M. Rieger, M. Rovere, H. Sakulin, J. Salfeld-Nebgen, S. Scarfi, C. Schäfer, C. Schwick, M. Selvaggi, A. Sharma, P. Silva, W. Snoeys, P. Sphicas, S. Summers, V. R. Tavolaro, D. Treille, A. Tsirou, G. P. Van Onsem, M. Verzetti, J. Wanczyk, K. A. Wozniak, W. D. Zeuner, L. Caminada, A. Ebrahimi, W. Erdmann, R. Horisberger, Q. Ingram, H. C. Kaestli, D. Kotlinski, U. Langenegger, M. Missiroli, T. Rohe, K. Androsov, M. Backhaus, P. Berger, A. Calandri, N. Chernyavskaya, A. De Cosa, G. Dissertori, M. Dittmar, M. Donegà, C. Dorfer, F. Eble, F. Glessgen, T. A. Gómez Espinosa, C. Grab, D. Hits, W. Lustermann, A.-M. Lyon, R. A. Manzoni, C. Martin Perez, M. T. Meinhard, F. Micheli, F. Nessi-Tedaldi, J. Niedziela, F. Pauss, V. Perovic, G. Perrin, S. Pigazzini, M. G. Ratti, M. Reichmann, C. Reissel, T. Reitenspiess, B. Ristic, D. Ruini, D. A. Sanz Becerra, M. Schönenberger, V. Stampf, J. Steggemann, R. Wallny, D. H. Zhu, C. Amsler, P. Bärtschi, C. Botta, D. Brzhechko, M. F. Canelli, K. Cormier, A. De Wit, R. Del Burgo, J. K. Heikkilä, M. Huwiler, A. Jofrehei, B. Kilminster, S. Leontsinis, A. Macchiolo, P. Meiring, V. M. Mikuni, U. Molinatti, I. Neutelings, A. Reimers, P. Robmann, S. Sanchez Cruz, K. Schweiger, Y. Takahashi, C. Adloff, C. M. Kuo, W. Lin, A. Roy, T. Sarkar, S. S. Yu, L. Ceard, Y. Chao, K. F. Chen, P. H. Chen, W.-S. Hou, Y. Y. Li, R.-S. Lu, E. Paganis, A. Psallidas, A. Steen, H. y. Wu, E. Yazgan, P. R. Yu, B. Asavapibhop, C. Asawatangtrakuldee, N. Srimanobhas, F. Boran, S. Damarseckin, Z. S. Demiroglu, F. Dolek, I. Dumanoglu, E. Eskut, Y. Guler, E. Gurpinar Guler, I. Hos, C. Isik, O. Kara, A. Kayis Topaksu, U. Kiminsu, G. Onengut, K. Ozdemir, A. Polatoz, A. E. Simsek, B. Tali, U. G. Tok, S. Turkcapar, I. S. Zorbakir, C. Zorbilmez, B. Isildak, G. Karapinar, K. Ocalan, M. Yalvac, B. Akgun, I. O. Atakisi, E. Gülmez, M. Kaya, O. Kaya, Ö. Özçelik, S. Tekten, E. A. Yetkin, A. Cakir, K. Cankocak, Y. Komurcu, S. Sen, S. Cerci, B. Kaynak, S. Ozkorucuklu, D. Sunar Cerci, B. Grynyov, L. Levchuk, D. Anthony, E. Bhal, S. Bologna, J. J. Brooke, A. Bundock, E. Clement, D. Cussans, H. Flacher, J. Goldstein, G. P. Heath, H. F. Heath, L. Kreczko, B. Krikler, S. Paramesvaran, S. Seif El Nasr-Storey, V. J. Smith, N. Stylianou, R. White, K. W. Bell, A. Belyaev, C. Brew, R. M. Brown, D. J. A. Cockerill, K. V. Ellis, K. Harder, S. Harper, J. Linacre, K. Manolopoulos, D. M. Newbold, E. Olaiya, D. Petyt, T. Reis, T. Schuh, C. H. Shepherd-Themistocleous, I. R. Tomalin, T. Williams, R. Bainbridge, P. Bloch, S. Bonomally, J. Borg, S. Breeze, O. Buchmuller, V. Cepaitis, G. S. Chahal, D. Colling, P. Dauncey, G. Davies, M. Della Negra, S. Fayer, G. Fedi, G. Hall, M. H. Hassanshahi, G. Iles, J. Langford, L. Lyons, A.-M. Magnan, S. Malik, A. Martelli, D. G. Monk, J. Nash, M. Pesaresi, D. M. Raymond, A. Richards, A. Rose, E. Scott, C. Seez, A. Shtipliyski, A. Tapper, K. Uchida, T. Virdee, M. Vojinovic, N. Wardle, S. N. Webb, D. Winterbottom, A. G. Zecchinelli, K. Coldham, J. E. Cole, A. Khan, P. Kyberd, I. D. Reid, L. Teodorescu, S. Zahid, S. Abdullin, A. Brinkerhoff, B. Caraway, J. Dittmann, K. Hatakeyama, A. R. Kanuganti, B. McMaster, N. Pastika, S. Sawant, C. Sutantawibul, J. Wilson, R. Bartek, A. Dominguez, R. Uniyal, A. M. Vargas Hernandez, A. Buccilli, S. I. Cooper, D. Di Croce, S. V. Gleyzer, C. Henderson, C. U. Perez, P. Rumerio, C. West, A. Akpinar, A. Albert, D. Arcaro, C. Cosby, Z. Demiragli, E. Fontanesi, D. Gastler, J. Rohlf, K. Salyer, D. Sperka, D. Spitzbart, I. Suarez, A. Tsatsos, S. Yuan, D. Zou, G. Benelli, B. Burkle, X. Coubez, D. Cutts, M. Hadley, U. Heintz, J. M. Hogan, G. Landsberg, K. T. Lau, M. Lukasik, J. Luo, M. Narain, S. Sagir, E. Usai, W. Y. Wong, X. Yan, D. Yu, W. Zhang, J. Bonilla, C. Brainerd, R. Breedon, M. Calderon De La Barca Sanchez, M. Chertok, J. Conway, P. T. Cox, R. Erbacher, G. Haza, F. Jensen, O. Kukral, R. Lander, M. Mulhearn, D. Pellett, B. Regnery, D. Taylor, Y. Yao, F. Zhang, M. Bachtis, R. Cousins, A. Datta, D. Hamilton, J. Hauser, M. Ignatenko, M. A. Iqbal, T. Lam, N. Mccoll, W. A. Nash, S. Regnard, D. Saltzberg, B. Stone, V. Valuev, K. Burt, Y. Chen, R. Clare, J. W. Gary, M. Gordon, G. Hanson, G. Karapostoli, O. R. Long, N. Manganelli, M. Olmedo Negrete, W. Si, S. Wimpenny, Y. Zhang, J. G. Branson, P. Chang, S. Cittolin, S. Cooperstein, N. Deelen, D. Diaz, J. Duarte, R. Gerosa, L. Giannini, D. Gilbert, J. Guiang, R. Kansal, V. Krutelyov, R. Lee, J. Letts, M. Masciovecchio, S. May, M. Pieri, B. V. Sathia Narayanan, V. Sharma, M. Tadel, A. Vartak, F. Würthwein, Y. Xiang, A. Yagil, N. Amin, C. Campagnari, M. Citron, A. Dorsett, V. Dutta, J. Incandela, M. Kilpatrick, J. Kim, B. Marsh, H. Mei, M. Oshiro, M. Quinnan, J. Richman, U. Sarica, D. Stuart, S. Wang, A. Bornheim, O. Cerri, I. Dutta, J. M. Lawhorn, N. Lu, J. Mao, H. B. Newman, J. Ngadiuba, T. Q. Nguyen, M. Spiropulu, J. R. Vlimant, C. Wang, S. Xie, Z. Zhang, R. Y. Zhu, J. Alison, S. An, M. B. Andrews, P. Bryant, T. Ferguson, A. Harilal, C. Liu, T. Mudholkar, M. Paulini, A. Sanchez, J. P. Cumalat, W. T. Ford, A. Hassani, E. MacDonald, R. Patel, A. Perloff, C. Savard, K. Stenson, K. A. Ulmer, S. R. Wagner, J. Alexander, S. Bright-thonney, Y. Cheng, D. J. Cranshaw, S. Hogan, J. Monroy, J. R. Patterson, D. Quach, J. Reichert, M. Reid, A. Ryd, W. Sun, J. Thom, P. Wittich, R. Zou, M. Albrow, M. Alyari, G. Apollinari, A. Apresyan, A. Apyan, S. Banerjee, L. A. T. Bauerdick, D. Berry, J. Berryhill, P. C. Bhat, K. Burkett, J. N. Butler, A. Canepa, G. B. Cerati, H. W. K. Cheung, F. Chlebana, M. Cremonesi, K. F. Di Petrillo, V. D. Elvira, Y. Feng, J. Freeman, Z. Gecse, L. Gray, D. Green, S. Grünendahl, O. Gutsche, R. M. Harris, R. Heller, T. C. Herwig, J. Hirschauer, B. Jayatilaka, S. Jindariani, M. Johnson, U. Joshi, T. Klijnsma, B. Klima, K. H. M. Kwok, S. Lammel, D. Lincoln, R. Lipton, T. Liu, C. Madrid, K. Maeshima, C. Mantilla, D. Mason, P. McBride, P. Merkel, S. Mrenna, S. Nahn, V. O’Dell, V. Papadimitriou, K. Pedro, C. Pena, O. Prokofyev, F. Ravera, A. Reinsvold Hall, L. Ristori, B. Schneider, E. Sexton-Kennedy, N. Smith, A. Soha, W. J. Spalding, L. Spiegel, S. Stoynev, J. Strait, L. Taylor, S. Tkaczyk, N. V. Tran, L. Uplegger, E. W. Vaandering, H. A. Weber, D. Acosta, P. Avery, D. Bourilkov, L. Cadamuro, V. Cherepanov, F. Errico, R. D. Field, D. Guerrero, B. M. Joshi, M. Kim, E. Koenig, J. Konigsberg, A. Korytov, K. H. Lo, K. Matchev, N. Menendez, G. Mitselmakher, A. Muthirakalayil Madhu, N. Rawal, D. Rosenzweig, S. Rosenzweig, K. Shi, J. Sturdy, J. Wang, E. Yigitbasi, X. Zuo, T. Adams, A. Askew, R. Habibullah, V. Hagopian, K. F. Johnson, R. Khurana, T. Kolberg, G. Martinez, H. Prosper, C. Schiber, R. Yohay, J. Zhang, M. M. Baarmand, S. Butalla, T. Elkafrawy, M. Hohlmann, R. Kumar Verma, D. Noonan, M. Rahmani, M. Saunders, F. Yumiceva, M. R. Adams, H. Becerril Gonzalez, R. Cavanaugh, X. Chen, S. Dittmer, O. Evdokimov, C. E. Gerber, D. A. Hangal, D. J. Hofman, A. H. Merrit, C. Mills, G. Oh, T. Roy, S. Rudrabhatla, M. B. Tonjes, N. Varelas, J. Viinikainen, X. Wang, Z. Wu, Z. Ye, M. Alhusseini, K. Dilsiz, R. P. Gandrajula, O. K. Köseyan, J.-P. Merlo, A. Mestvirishvili, J. Nachtman, H. Ogul, Y. Onel, A. Penzo, C. Snyder, E. Tiras, O. Amram, B. Blumenfeld, L. Corcodilos, J. Davis, M. Eminizer, A. V. Gritsan, S. Kyriacou, P. Maksimovic, J. Roskes, M. Swartz, T. Á. Vámi, A. Abreu, J. Anguiano, C. Baldenegro Barrera, P. Baringer, A. Bean, A. Bylinkin, Z. Flowers, T. Isidori, S. Khalil, J. King, G. Krintiras, A. Kropivnitskaya, M. Lazarovits, C. Lindsey, J. Marquez, N. Minafra, M. Murray, M. Nickel, C. Rogan, C. Royon, R. Salvatico, S. Sanders, E. Schmitz, C. Smith, J. D. Tapia Takaki, Q. Wang, Z. Warner, J. Williams, G. Wilson, S. Duric, A. Ivanov, K. Kaadze, D. Kim, Y. Maravin, T. Mitchell, A. Modak, K. Nam, F. Rebassoo, D. Wright, E. Adams, A. Baden, O. Baron, A. Belloni, S. C. Eno, N. J. Hadley, S. Jabeen, R. G. Kellogg, T. Koeth, A. C. Mignerey, S. Nabili, M. Seidel, A. Skuja, L. Wang, K. Wong, D. Abercrombie, G. Andreassi, R. Bi, S. Brandt, W. Busza, I. A. Cali, Y. Chen, M. D’Alfonso, J. Eysermans, G. Gomez Ceballos, M. Goncharov, P. Harris, M. Hu, M. Klute, D. Kovalskyi, J. Krupa, Y.-J. Lee, B. Maier, C. Mironov, C. Paus, D. Rankin, C. Roland, G. Roland, Z. Shi, G. S. F. Stephans, K. Tatar, J. Wang, Z. Wang, B. Wyslouch, R. M. Chatterjee, A. Evans, P. Hansen, J. Hiltbrand, Sh. Jain, M. Krohn, Y. Kubota, J. Mans, M. Revering, R. Rusack, R. Saradhy, N. Schroeder, N. Strobbe, M. A. Wadud, K. Bloom, M. Bryson, S. Chauhan, D. R. Claes, C. Fangmeier, L. Finco, F. Golf, J. R. González Fernández, C. Joo, I. Kravchenko, M. Musich, I. Reed, J. E. Siado, G. R. Snow, W. Tabb, F. Yan, G. Agarwal, H. Bandyopadhyay, L. Hay, I. Iashvili, A. Kharchilava, C. McLean, D. Nguyen, J. Pekkanen, S. Rappoccio, A. Williams, G. Alverson, E. Barberis, C. Freer, Y. Haddad, A. Hortiangtham, J. Li, G. Madigan, B. Marzocchi, D. M. Morse, V. Nguyen, T. Orimoto, A. Parker, L. Skinnari, A. Tishelman-Charny, T. Wamorkar, B. Wang, A. Wisecarver, D. Wood, S. Bhattacharya, J. Bueghly, Z. Chen, A. Gilbert, T. Gunter, K. A. Hahn, N. Odell, M. H. Schmitt, M. Velasco, R. Band, R. Bucci, A. Das, N. Dev, R. Goldouzian, M. Hildreth, K. Hurtado Anampa, C. Jessop, K. Lannon, J. Lawrence, N. Loukas, N. Marinelli, I. Mcalister, T. McCauley, F. Meng, K. Mohrman, Y. Musienko, R. Ruchti, P. Siddireddy, M. Wayne, A. Wightman, M. Wolf, M. Zarucki, L. Zygala, B. Bylsma, B. Cardwell, L. S. Durkin, B. Francis, C. Hill, M. Nunez Ornelas, K. Wei, B. L. Winer, B. R. Yates, F. M. Addesa, B. Bonham, P. Das, G. Dezoort, P. Elmer, A. Frankenthal, B. Greenberg, N. Haubrich, S. Higginbotham, A. Kalogeropoulos, G. Kopp, S. Kwan, D. Lange, M. T. Lucchini, D. Marlow, K. Mei, I. Ojalvo, J. Olsen, C. Palmer, D. Stickland, C. Tully, S. Malik, S. Norberg, A. S. Bakshi, V. E. Barnes, R. Chawla, S. Das, L. Gutay, M. Jones, A. W. Jung, S. Karmarkar, M. Liu, G. Negro, N. Neumeister, G. Paspalaki, C. C. Peng, S. Piperov, A. Purohit, J. F. Schulte, M. Stojanovic, J. Thieman, F. Wang, R. Xiao, W. Xie, J. Dolen, N. Parashar, A. Baty, M. Decaro, S. Dildick, K. M. Ecklund, S. Freed, P. Gardner, F. J. M. Geurts, A. Kumar, W. Li, B. P. Padley, R. Redjimi, W. Shi, A. G. Stahl Leiton, S. Yang, L. Zhang, Y. Zhang, A. Bodek, P. de Barbaro, R. Demina, J. L. Dulemba, C. Fallon, T. Ferbel, M. Galanti, A. Garcia-Bellido, O. Hindrichs, A. Khukhunaishvili, E. Ranken, R. Taus, B. Chiarito, J. P. Chou, A. Gandrakota, Y. Gershtein, E. Halkiadakis, A. Hart, M. Heindl, E. Hughes, S. Kaplan, O. Karacheban, I. Laflotte, A. Lath, R. Montalvo, K. Nash, M. Osherson, S. Salur, S. Schnetzer, S. Somalwar, R. Stone, S. A. Thayil, S. Thomas, H. Wang, H. Acharya, A. G. Delannoy, S. Spanier, O. Bouhali, M. Dalchenko, A. Delgado, R. Eusebi, J. Gilmore, T. Huang, T. Kamon, H. Kim, S. Luo, S. Malhotra, R. Mueller, D. Overton, D. Rathjens, A. Safonov, N. Akchurin, J. Damgov, V. Hegde, S. Kunori, K. Lamichhane, S. W. Lee, T. Mengke, S. Muthumuni, T. Peltola, I. Volobouev, Z. Wang, A. Whitbeck, E. Appelt, S. Greene, A. Gurrola, W. Johns, A. Melo, H. Ni, K. Padeken, F. Romeo, P. Sheldon, S. Tuo, J. Velkovska, M. W. Arenton, B. Cox, G. Cummings, J. Hakala, R. Hirosky, M. Joyce, A. Ledovskoy, A. Li, C. Neu, B. Tannenwald, S. White, E. Wolfe, N. Poudyal, K. Black, T. Bose, J. Buchanan, C. Caillol, S. Dasu, I. De Bruyn, P. Everaerts, F. Fienga, C. Galloni, H. He, M. Herndon, A. Hervé, U. Hussain, A. Lanaro, A. Loeliger, R. Loveless, J. Madhusudanan Sreekala, A. Mallampalli, A. Mohammadi, D. Pinna, A. Savin, V. Shang, V. Sharma, W. H. Smith, D. Teague, S. Trembath-reichert, W. Vetens, CMS Collaboration

**Affiliations:** 1 Yerevan Physics Institute, Yerevan, Armenia; 2 Institut für Hochenergiephysik, Vienna, Austria; 3 Institute for Nuclear Problems, Minsk, Belarus; 4 Universiteit Antwerpen, Antwerp, Belgium; 5 Vrije Universiteit Brussel, Brussels, Belgium; 6 Université Libre de Bruxelles, Brussels, Belgium; 7 Ghent University, Ghent, Belgium; 8 Université Catholique de Louvain, Louvain-la-Neuve, Belgium; 9 Centro Brasileiro de Pesquisas Fisicas, Rio de Janeiro, Brazil; 10 Universidade do Estado do Rio de Janeiro, Rio de Janeiro, Brazil; 11 Universidade Estadual Paulista, Universidade Federal do ABC, São Paulo, Brazil; 12 Institute for Nuclear Research and Nuclear Energy, Bulgarian Academy of Sciences, Sofia, Bulgaria; 13 University of Sofia, Sofia, Bulgaria; 14 Beihang University, Beijing, China; 15 Department of Physics, Tsinghua University, Beijing, China; 16 Institute of High Energy Physics, Beijing, China; 17 State Key Laboratory of Nuclear Physics and Technology, Peking University, Beijing, China; 18 Sun Yat-Sen University, Guangzhou, China; 19 Institute of Modern Physics and Key Laboratory of Nuclear Physics and Ion-beam Application (MOE), Fudan University, Shanghai, China; 20 Zhejiang University, Hangzhou, China; 21 Universidad de Los Andes, Bogotá, Colombia; 22 Universidad de Antioquia, Medellín, Colombia; 23 Faculty of Electrical Engineering, Mechanical Engineering and Naval Architecture, University of Split, Split, Croatia; 24 University of Split, Faculty of Science, Split, Croatia; 25 Institute Rudjer Boskovic, Zagreb, Croatia; 26 University of Cyprus, Nicosia, Cyprus; 27 Charles University, Prague, Czech Republic; 28 Escuela Politecnica Nacional, Quito, Ecuador; 29 Universidad San Francisco de Quito, Quito, Ecuador; 30 Academy of Scientific Research and Technology of the Arab Republic of Egypt, Egyptian Network of High Energy Physics, Cairo, Egypt; 31 Center for High Energy Physics (CHEP-FU), Fayoum University, El-Fayoum, Egypt; 32 National Institute of Chemical Physics and Biophysics, Tallinn, Estonia; 33 Department of Physics, University of Helsinki, Helsinki, Finland; 34 Helsinki Institute of Physics, Helsinki, Finland; 35 Lappeenranta University of Technology, Lappeenranta, Finland; 36 IRFU, CEA, Université Paris-Saclay, Gif-sur-Yvette, France; 37 Laboratoire Leprince-Ringuet, CNRS/IN2P3, Ecole Polytechnique, Institut Polytechnique de Paris, Palaiseau, France; 38 Université de Strasbourg, CNRS, IPHC UMR 7178, Strasbourg, France; 39 Institut de Physique des 2 Infinis de Lyon (IP2I), Villeurbanne, France; 40 Georgian Technical University, Tbilisi, Georgia; 41 I. Physikalisches Institut, RWTH Aachen University, Aachen, Germany; 42 III. Physikalisches Institut A, RWTH Aachen University, Aachen, Germany; 43 III. Physikalisches Institut B, RWTH Aachen University, Aachen, Germany; 44 Deutsches Elektronen-Synchrotron, Hamburg, Germany; 45 University of Hamburg, Hamburg, Germany; 46 Karlsruher Institut fuer Technologie, Karlsruhe, Germany; 47 Institute of Nuclear and Particle Physics (INPP), NCSR Demokritos, Aghia Paraskevi, Greece; 48 National and Kapodistrian University of Athens, Athens, Greece; 49 National Technical University of Athens, Athens, Greece; 50 University of Ioánnina, Ioannina, Greece; 51 MTA-ELTE Lendület CMS Particle and Nuclear Physics Group, Eötvös Loránd University, Budapest, Hungary; 52 Wigner Research Centre for Physics, Budapest, Hungary; 53 Institute of Nuclear Research ATOMKI, Debrecen, Hungary; 54 Institute of Physics, University of Debrecen, Debrecen, Hungary; 55 Eszterhazy Karoly University, Karoly Robert Campus, Gyongyos, Hungary; 56 Indian Institute of Science (IISc), Bangalore, India; 57 National Institute of Science Education and Research, HBNI, Bhubaneswar, India; 58 Panjab University, Chandigarh, India; 59 University of Delhi, Delhi, India; 60 Saha Institute of Nuclear Physics, HBNI, Kolkata, India; 61 Indian Institute of Technology Madras, Chennai, India; 62 Bhabha Atomic Research Centre, Mumbai, India; 63 Tata Institute of Fundamental Research-A, Mumbai, India; 64 Tata Institute of Fundamental Research-B, Mumbai, India; 65 Indian Institute of Science Education and Research (IISER), Pune, India; 66 Department of Physics, Isfahan University of Technology, Isfahan, Iran; 67 Institute for Research in Fundamental Sciences (IPM), Tehran, Iran; 68 University College Dublin, Dublin, Ireland; 69 INFN Sezione di Bari, Universit’a di Bari, Politecnico di Bari, Bari, Italy; 70 INFN Sezione di Bologna, Università di Bologna, Bologna, Italy; 71 INFN Sezione di Catania, Università di Catania, Catania, Italy; 72 INFN Sezione di Firenze, Università di Firenze, Florence, Italy; 73 INFN Laboratori Nazionali di Frascati, Frascati, Italy; 74 INFN Sezione di Genova, Università di Genova, Genoa, Italy; 75 INFN Sezione di Milano-Bicocca, Università di Milano-Bicocca, Milan, Italy; 76 INFN Sezione di Napoli, Università di Napoli ‘Federico II’, Napoli, Italy, Università della Basilicata, Potenza, Italy, Università G. Marconi, Rome, Italy; 77 INFN Sezione di Padova, Università di Padova, Padua, Italy, Università di Trento, Trento, Italy; 78 INFN Sezione di Pavia, Università di Pavia, Pavia, Italy; 79 INFN Sezione di Perugia, Università di Perugia, Perugia, Italy; 80 INFN Sezione di Pisa, Università di Pisa, Scuola Normale Superiore di Pisa, Pisa, Italy, Università di Siena, Siena, Italy; 81 INFN Sezione di Roma, Sapienza Università di Roma, Rome, Italy; 82 INFN Sezione di Torino, Università di Torino, Turin, Italy, Università del Piemonte Orientale, Novara, Italy; 83 INFN Sezione di Trieste, Università di Trieste, Trieste, Italy; 84 Kyungpook National University, Daegu, Korea; 85 Chonnam National University, Institute for Universe and Elementary Particles, Kwangju, Korea; 86 Hanyang University, Seoul, Korea; 87 Korea University, Seoul, Korea; 88 Kyung Hee University, Department of Physics, Seoul, Republic of Korea; 89 Sejong University, Seoul, Korea; 90 Seoul National University, Seoul, Korea; 91 University of Seoul, Seoul, Korea; 92 Department of Physics, Yonsei University, Seoul, Korea; 93 Sungkyunkwan University, Suwon, Korea; 94 College of Engineering and Technology, American University of the Middle East (AUM), Egaila, Kuwait; 95 Riga Technical University, Riga, Latvia; 96 Vilnius University, Vilnius, Lithuania; 97 National Centre for Particle Physics, Universiti Malaya, Kuala Lumpur, Malaysia; 98 Universidad de Sonora (UNISON), Hermosillo, Mexico; 99 Centro de Investigacion y de Estudios Avanzados del IPN, Mexico City, Mexico; 100 Universidad Iberoamericana, Mexico City, Mexico; 101 Benemerita Universidad Autonoma de Puebla, Puebla, Mexico; 102 University of Montenegro, Podgorica, Montenegro; 103 University of Auckland, Auckland, New Zealand; 104 University of Canterbury, Christchurch, New Zealand; 105 National Centre for Physics, Quaid-I-Azam University, Islamabad, Pakistan; 106 Faculty of Computer Science, Electronics and Telecommunications, AGH University of Science and Technology, Kraków, Poland; 107 National Centre for Nuclear Research, Swierk, Poland; 108 Institute of Experimental Physics, Faculty of Physics, University of Warsaw, Warsaw, Poland; 109 Laboratório de Instrumentação e Física Experimental de Partículas, Lisbon, Portugal; 110 Joint Institute for Nuclear Research, Dubna, Russia; 111 Petersburg Nuclear Physics Institute, Gatchina (St. Petersburg), Russia; 112 Institute for Nuclear Research, Moscow, Russia; 113 Institute for Theoretical and Experimental Physics named by A.I. Alikhanov of NRC ‘Kurchatov Institute’, Moscow, Russia; 114 Moscow Institute of Physics and Technology, Moscow, Russia; 115 National Research Nuclear University ‘Moscow Engineering Physics Institute’ (MEPhI), Moscow, Russia; 116 P.N. Lebedev Physical Institute, Moscow, Russia; 117 Skobeltsyn Institute of Nuclear Physics, Lomonosov Moscow State University, Moscow, Russia; 118 Novosibirsk State University (NSU), Novosibirsk, Russia; 119 Institute for High Energy Physics of National Research Centre ‘Kurchatov Institute’, Protvino, Russia; 120 National Research Tomsk Polytechnic University, Tomsk, Russia; 121 Tomsk State University, Tomsk, Russia; 122 Faculty of Physics and VINCA Institute of Nuclear Sciences, University of Belgrade, Belgrade, Serbia; 123 Centro de Investigaciones Energéticas Medioambientales y Tecnológicas (CIEMAT), Madrid, Spain; 124 Universidad Autónoma de Madrid, Madrid, Spain; 125 Instituto Universitario de Ciencias y Tecnologías Espaciales de Asturias (ICTEA), Universidad de Oviedo, Oviedo, Spain; 126 Instituto de Física de Cantabria (IFCA), CSIC-Universidad de Cantabria, Santander, Spain; 127 University of Colombo, Colombo, Sri Lanka; 128 Department of Physics, University of Ruhuna, Matara, Sri Lanka; 129 CERN, European Organization for Nuclear Research, Geneva, Switzerland; 130 Paul Scherrer Institut, Villigen, Switzerland; 131 ETH Zurich-Institute for Particle Physics and Astrophysics (IPA), Zurich, Switzerland; 132 Universität Zürich, Zurich, Switzerland; 133 National Central University, Chung-Li, Taiwan; 134 National Taiwan University (NTU), Taipei, Taiwan; 135 Department of Physics, Faculty of Science, Chulalongkorn University, Bangkok, Thailand; 136 Physics Department, Science and Art Faculty, Çukurova University, Adana, Turkey; 137 Physics Department, Middle East Technical University, Ankara, Turkey; 138 Bogazici University, Istanbul, Turkey; 139 Istanbul Technical University, Istanbul, Turkey; 140 Istanbul University, Istanbul, Turkey; 141 Institute for Scintillation Materials of National Academy of Science of Ukraine, Kharkov, Ukraine; 142 National Scientific Center, Kharkov Institute of Physics and Technology, Kharkov, Ukraine; 143 University of Bristol, Bristol, UK; 144 Rutherford Appleton Laboratory, Didcot, UK; 145 Imperial College, London, UK; 146 Brunel University, Uxbridge, UK; 147 Baylor University, Waco, USA; 148 Catholic University of America, Washington, DC, USA; 149 The University of Alabama, Tuscaloosa, USA; 150 Boston University, Boston, USA; 151 Brown University, Providence, USA; 152 University of California, Davis, Davis, USA; 153 University of California, Los Angeles, USA; 154 University of California, Riverside, Riverside, USA; 155 University of California, San Diego, La Jolla, USA; 156 Department of Physics, University of California, Santa Barbara, Santa Barbara, USA; 157 California Institute of Technology, Pasadena, USA; 158 Carnegie Mellon University, Pittsburgh, USA; 159 University of Colorado Boulder, Boulder, USA; 160 Cornell University, Ithaca, USA; 161 Fermi National Accelerator Laboratory, Batavia, USA; 162 University of Florida, Gainesville, USA; 163 Florida State University, Tallahassee, USA; 164 Florida Institute of Technology, Melbourne, USA; 165 University of Illinois at Chicago (UIC), Chicago, USA; 166 The University of Iowa, Iowa City, USA; 167 Johns Hopkins University, Baltimore, USA; 168 The University of Kansas, Lawrence, USA; 169 Kansas State University, Manhattan, USA; 170 Lawrence Livermore National Laboratory, Livermore, USA; 171 University of Maryland, College Park, USA; 172 Massachusetts Institute of Technology, Cambridge, USA; 173 University of Minnesota, Minneapolis, USA; 174 University of Nebraska-Lincoln, Lincoln, USA; 175 State University of New York at Buffalo, Buffalo, USA; 176 Northeastern University, Boston, USA; 177 Northwestern University, Evanston, USA; 178 University of Notre Dame, Notre Dame, USA; 179 The Ohio State University, Columbus, USA; 180 Princeton University, Princeton, USA; 181 University of Puerto Rico, Mayagüez, USA; 182 Purdue University, West Lafayette, USA; 183 Purdue University Northwest, Hammond, USA; 184 Rice University, Houston, USA; 185 University of Rochester, Rochester, USA; 186 Rutgers, The State University of New Jersey, Piscataway, USA; 187 University of Tennessee, Knoxville, USA; 188 Texas A&M University, College Station, USA; 189 Texas Tech University, Lubbock, USA; 190 Vanderbilt University, Nashville, USA; 191 University of Virginia, Charlottesville, USA; 192 Wayne State University, Detroit, USA; 193 University of Wisconsin-Madison, Madison, WI USA; 194 Vienna University of Technology, Vienna, Austria; 195 Institute of Basic and Applied Sciences, Faculty of Engineering, Arab Academy for Science, Technology and Maritime Transport, Alexandria, Egypt; 196 Université Libre de Bruxelles, Brussels, Belgium; 197 Universidade Estadual de Campinas, Campinas, Brazil; 198 Federal University of Rio Grande do Sul, Porto Alegre, Brazil; 199 University of Chinese Academy of Sciences, Beijing, China; 200 Department of Physics, Tsinghua University, Beijing, China; 201 UFMS, Nova Andradina, Brazil; 202 Department of Physics, Nanjing Normal University, Nanjing, China; 203 The University of Iowa, Iowa City, USA; 204 Institute for Theoretical and Experimental Physics named by A.I. Alikhanov of NRC ‘Kurchatov Institute’, Moscow, Russia; 205 Joint Institute for Nuclear Research, Dubna, Russia; 206 Cairo University, Cairo, Egypt; 207 Helwan University, Cairo, Egypt; 208 Zewail City of Science and Technology, Zewail, Egypt; 209 Purdue University, West Lafayette, USA; 210 Université de Haute Alsace, Mulhouse, France; 211 Erzincan Binali Yildirim University, Erzincan, Turkey; 212 CERN, European Organization for Nuclear Research, Geneva, Switzerland; 213 III. Physikalisches Institut A, RWTH Aachen University, Aachen, Germany; 214 University of Hamburg, Hamburg, Germany; 215 Department of Physics, Isfahan University of Technology, Isfahan, Iran; 216 Brandenburg University of Technology, Cottbus, Germany; 217 Skobeltsyn Institute of Nuclear Physics, Lomonosov Moscow State University, Moscow, Russia; 218 Physics Department, Faculty of Science, Assiut University, Assiut, Egypt; 219 Eszterhazy Karoly University, Karoly Robert Campus, Gyongyos, Hungary; 220 Institute of Physics, University of Debrecen, Debrecen, Hungary; 221 Institute of Nuclear Research ATOMKI, Debrecen, Hungary; 222 MTA-ELTE Lendület CMS Particle and Nuclear Physics Group, Eötvös Loránd University, Budapest, Hungary; 223 Wigner Research Centre for Physics, Budapest, Hungary; 224 IIT Bhubaneswar, Bhubaneswar, India; 225 Institute of Physics, Bhubaneswar, India; 226 G.H.G. Khalsa College, Punjab, India; 227 Shoolini University, Solan, India; 228 University of Hyderabad, Hyderabad, India; 229 University of Visva-Bharati, Santiniketan, India; 230 Indian Institute of Technology (IIT), Mumbai, India; 231 Deutsches Elektronen-Synchrotron, Hamburg, Germany; 232 Sharif University of Technology, Tehran, Iran; 233 Department of Physics, University of Science and Technology of Mazandaran, Behshahr, Iran; 234 INFN Sezione di Bari, Università di Bari, Politecnico di Bari, Bari, Italy; 235 Italian National Agency for New Technologies, Energy and Sustainable Economic Development, Bologna, Italy; 236 Centro Siciliano di Fisica Nucleare e di Struttura Della Materia, Catania, Italy; 237 Università di Napoli ‘Federico II’, Naples, Italy; 238 Riga Technical University, Riga, Latvia; 239 Consejo Nacional de Ciencia y Tecnología, Mexico City, Mexico; 240 IRFU, CEA, Université Paris-Saclay, Gif-sur-Yvette, France; 241 Institute for Nuclear Research, Moscow, Russia; 242 National Research Nuclear University ‘Moscow Engineering Physics Institute’ (MEPhI), Moscow, Russia; 243 Institute of Nuclear Physics of the Uzbekistan Academy of Sciences, Tashkent, Uzbekistan; 244 St. Petersburg State Polytechnical University, St. Petersburg, Russia; 245 University of Florida, Gainesville, USA; 246 Imperial College, London, UK; 247 Moscow Institute of Physics and Technology, Moscow, Russia; 248 P.N. Lebedev Physical Institute, Moscow, Russia; 249 California Institute of Technology, Pasadena, USA; 250 Budker Institute of Nuclear Physics, Novosibirsk, Russia; 251 Faculty of Physics, University of Belgrade, Belgrade, Serbia; 252 Trincomalee Campus, Eastern University, Sri Lanka, Nilaveli, Sri Lanka; 253 INFN Sezione di Pavia, Università di Pavia, Pavia, Italy; 254 National and Kapodistrian University of Athens, Athens, Greece; 255 Ecole Polytechnique Fédérale Lausanne, Lausanne, Switzerland; 256 Universität Zürich, Zurich, Switzerland; 257 Stefan Meyer Institute for Subatomic Physics, Vienna, Austria; 258 Laboratoire d’Annecy-le-Vieux de Physique des Particules, IN2P3-CNRS, Annecy-le-Vieux, France; 259 Şırnak University, Sirnak, Turkey; 260 Near East University, Research Center of Experimental Health Science, Nicosia, Turkey; 261 Konya Technical University, Konya, Turkey; 262 Faculty of Engineering, Istanbul University-Cerrahpasa, Istanbul, Turkey; 263 Piri Reis University, Istanbul, Turkey; 264 Adiyaman University, Adiyaman, Turkey; 265 Ozyegin University, Istanbul, Turkey; 266 Izmir Institute of Technology, Izmir, Turkey; 267 Necmettin Erbakan University, Konya, Turkey; 268 Bozok Universitetesi Rektörlügü, Yozgat, Turkey; 269 Marmara University, Istanbul, Turkey; 270 Milli Savunma University, Istanbul, Turkey; 271 Kafkas University, Kars, Turkey; 272 Istanbul Bilgi University, Istanbul, Turkey; 273 Hacettepe University, Ankara, Turkey; 274 Vrije Universiteit Brussel, Brussels, Belgium; 275 School of Physics and Astronomy, University of Southampton, Southampton, UK; 276 IPPP Durham University, Durham, UK; 277 Faculty of Science, Monash University, Clayton, Australia; 278 Università di Torino, Turin, Italy; 279 Bethel University, St. Paul, Minneapolis, USA; 280 Karamanoğlu Mehmetbey University, Karaman, Turkey; 281 Ain Shams University, Cairo, Egypt; 282 Bingol University, Bingol, Turkey; 283 Georgian Technical University, Tbilisi, Georgia; 284 Sinop University, Sinop, Turkey; 285 Erciyes University, Kayseri, Turkey; 286 Texas A&M University at Qatar, Doha, Qatar; 287 Kyungpook National University, Daegu, Korea; 288 CERN, 1211 Geneva 23, Switzerland

## Abstract

A search for charged Higgs bosons produced in vector boson fusion processes and decaying into vector bosons, using proton–proton collisions at $\sqrt{s}=13\,{\text {TeV}}$ at the LHC, is reported. The data sample corresponds to an integrated luminosity of 137$\,{\text {fb}}^{-1}$ collected with the CMS detector. Events are selected by requiring two or three electrons or muons, moderate missing transverse momentum, and two jets with a large rapidity separation and a large dijet mass. No excess of events with respect to the standard model background predictions is observed. Model independent upper limits at 95% confidence level are reported on the product of the cross section and branching fraction for vector boson fusion production of charged Higgs bosons as a function of mass, from 200 to 3000$\,{\text {GeV}}$. The results are interpreted in the context of the Georgi–Machacek model.

## Introduction

The discovery [1–3] of a Higgs boson [4–9] at the CERN LHC marks an important milestone in the exploration of the electroweak (EW) sector of the standard model (SM) of particle physics. Measurements of vector boson scattering (VBS) processes at the LHC may reveal hints for extensions of the SM. In particular, extended Higgs sectors with additional SU(2) doublets [10–13] or triplets [14–19] introduce couplings of gauge bosons to heavy neutral or charged Higgs bosons with specific signatures like singly or doubly charged Higgs boson decays to ${\text {W}}{\text {Z}}$ boson pairs or same-sign ${\text {W}}^\pm {\text {W}}^\pm$ boson pairs, respectively.

At the LHC, interactions from VBS are characterized by the presence of two gauge bosons in association with two forward jets with a large pseudorapidity separation ($|\Delta \eta _{\mathrm {j}\mathrm {j}} |$) and a large dijet invariant mass ($m_{\mathrm {j}\mathrm {j}}$). An excess of events with respect to the SM predictions could indicate the presence of new resonances, such as singly or doubly charged Higgs bosons. Extended Higgs sectors with additional SU(2) isotriplet scalars give rise to charged Higgs bosons with couplings to ${\text {W}}$ and ${\text {Z}}$ bosons at the tree-level [19]. Specifically, the Georgi–Machacek (GM) model [18, 20], with both real and complex triplets, preserves a global symmetry SU(2)$_\mathrm {L}\times$SU(2)$_\mathrm {R}$, which is broken by the Higgs vacuum expectation value to the diagonal subgroup SU(2)$_{\mathrm {L}+\mathrm {R}}$. Thus, the tree-level ratio of the ${\text {W}}$ and ${\text {Z}}$ boson masses is protected against large radiative corrections. In this model, singly (doubly) charged Higgs bosons that decay to ${\text {W}}$ and ${\text {Z}}$ bosons (same-sign ${\text {W}}$ boson pairs) are produced via vector boson fusion (VBF).

**Fig. 1:**
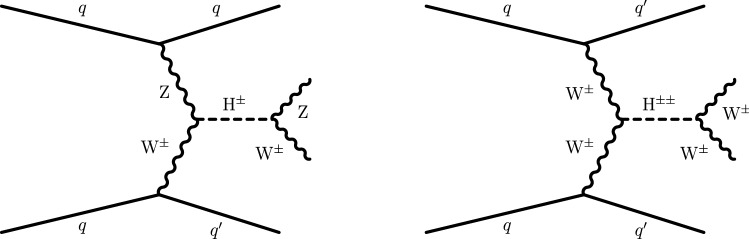
Examples of Feynman diagrams showing the production of singly (upper) and doubly (lower) charged Higgs bosons via VBF

The charged Higgs bosons ${\text {H}} ^{\pm }$ and ${\text {H}} ^{\pm \pm }$ in the GM model are degenerate in mass (denoted as $m_{{\text {H}} _{5}}$) at tree level and transform as a quintuplet under the SU(2)$_{\mathrm {L}+\mathrm {R}}$ symmetry. The ${\text {H}} ^{\pm }$ and ${\text {H}} ^{\pm \pm }$ bosons are also collectively referred to as ${\text {H}} _5$ in the context of the GM model. Production and decays of the ${\text {H}} _5$ states depend on the two parameters $m_{{\text {H}} _{5}}$ and $s_{{\text {H}}}$, where $s_{{\text {H}}}^2$ characterizes the fraction of the ${\text {W}}$ boson mass squared generated by the vacuum expectation value of the triplet fields. The ${\text {H}} _5$ states are fermiophobic and are assumed to decay to vector boson pairs with branching fraction of 100% [21]. Figure 1 shows representative Feynman diagrams for the production and decay of the charged Higgs bosons. There are additional charged Higgs bosons ${\text {H}} ^{\pm }$ predicted in the GM model that transform as a triplet under the SU(2)$_{\mathrm {L}+\mathrm {R}}$ symmetry. These ${\text {H}} ^{\pm }$ bosons have only fermionic couplings and are not considered here.

This paper presents a search for ${\text {H}} ^{\pm }$ and ${\text {H}} ^{\pm \pm }$ that are produced via VBF and decay to ${\text {W}}{\text {Z}}$ and ${\text {W}}^\pm {\text {W}}^\pm$ boson pairs, respectively, using proton–proton (${\text {p}}{\text {p}}$) collisions at $\sqrt{s}=13\,{\text {TeV}}$. The data sample corresponds to an integrated luminosity of $137 \pm 2{\,{\text {fb}}^{-1}}$ [22–24], collected with the CMS detector [25] in three separate LHC operating periods during 2016, 2017, and 2018. The three data sets are analyzed independently, with appropriate calibrations and corrections, to account for the various LHC running conditions and the performance of the CMS detector.

The ${\text {W}}^\pm {\text {W}}^\pm$ and ${\text {W}}{\text {Z}}$ channels are simultaneously studied by performing a binned maximum-likelihood fit of distributions sensitive to these processes, following the methods described in Ref. [26]. The searches for ${\text {H}} ^{\pm }$ and ${\text {H}} ^{\pm \pm }$ are performed in the leptonic decay modes ${\text {W}}^\pm {\text {Z}}\rightarrow \ell ^\pm {\upnu }\ell '^\pm \ell '^\mp$ and ${\text {W}}^\pm {\text {W}}^\pm \rightarrow \ell ^\pm {\upnu }\ell '^\pm {\upnu }$, where $\ell , \ell ' = {\text {e}}$, ${\upmu }$. Candidate events contain either two identified leptons of the same charge or three identified charged leptons with the total charge of ±1, moderate missing transverse momentum ($p_{{\mathrm {T}}} ^{\text {miss}}$), and two jets with large values of $|\Delta \eta _{\mathrm {j}\mathrm {j}} |$ and $m_{\mathrm {j}\mathrm {j}}$.

Model independent upper limits at 95% confidence level (${\text {CL}}$) are reported on the product of the cross section and branching fraction for vector boson fusion production of the ${\text {H}} ^{\pm }$ and ${\text {H}} ^{\pm \pm }$ bosons individually. The results are also interpreted in the context of the GM model including the simultaneous contributions of the ${\text {H}} ^{\pm }$ and ${\text {H}} ^{\pm \pm }$ bosons. Searches for charged Higgs bosons in these topologies have been performed by the CMS Collaboration at $13\,{\text {TeV}}$ using the data sample collected during 2016 [27–29]. The ATLAS and CMS Collaborations have also set constraints on the GM model by performing searches for charged Higgs bosons in semileptonic final states at $8\,{\text {TeV}}$ [30] and $13\,{\text {TeV}}$ [31], respectively.

## The CMS detector

The central feature of the CMS apparatus is a superconducting solenoid of 6$\,{\text {m}}$ internal diameter, providing a magnetic field of 3.8$\,{\text {T}}$. Within the solenoid volume are a silicon pixel and strip tracker, a lead-tungstate crystal electromagnetic calorimeter (ECAL), and a brass and scintillator hadron calorimeter, each composed of a barrel and two endcap sections. Forward calorimeters extend the $\eta$ coverage provided by the barrel and endcap detectors up to $|\eta |<5$. Muons are detected in gas-ionization chambers embedded in the steel magnetic flux-return yoke outside the solenoid. A more detailed description of the CMS detector, together with a definition of the coordinate system and the relevant kinematic variables, is reported in Ref. [25]. Events of interest are selected using a two-tiered trigger system [32]. The first level, composed of custom hardware processors, uses information from the calorimeters and muon detectors to select events at a rate of around 100$\,{\text {kHz}}$ within a fixed latency of 4$\,\upmu \text {s}$. The second level, known as the high-level trigger, consists of a farm of processors running a version of the full event reconstruction software optimized for fast processing, and reduces the event rate to around 1$\,{\text {kHz}}$ before data storage.

## Signal and background simulation

**Fig. 2:**
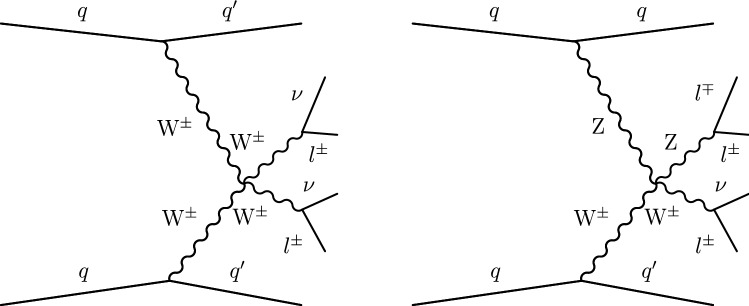
Representative Feynman diagrams of a VBS process contributing to the EW-induced production of events containing ${\text {W}}^\pm {\text {W}}^\pm$ (left) and ${\text {W}}{\text {Z}}$ (right) boson pairs decaying to leptons, and two forward jets

**Fig. 3:**
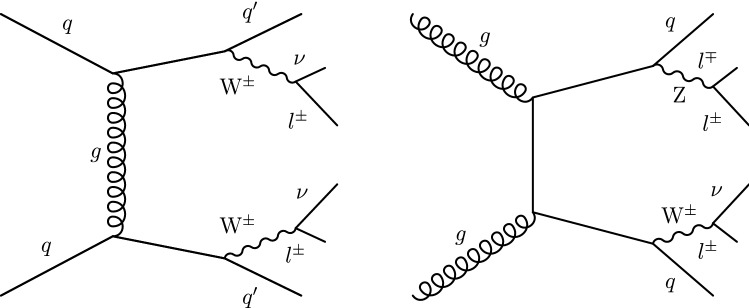
Representative Feynman diagrams of the QCD-induced production of ${\text {W}}^\pm {\text {W}}^\pm$ (left) and ${\text {W}}{\text {Z}}$ (right) boson pairs decaying to leptons, and two jets

Processes characterized by the presence of two gauge bosons in association with two forward jets are an important background contribution. The processes contributing to diboson plus two jets production that proceeds via the EW interaction are referred to as EW-induced diboson production, leading to tree-level contributions at $\mathcal {O}(\alpha ^4)$, where $\alpha$ is the EW coupling. Figure 2 shows representative Feynman diagrams of EW-induced diboson production involving quartic vertices. An additional contribution to the diboson plus two jets production arises via quantum chromodynamics (QCD) radiation, leading to tree-level contributions at $\mathcal {O}(\alpha ^2\alpha _{S}^2)$, where $\alpha _{S}$ is the strong coupling. This class of processes is referred to as QCD-induced diboson production. Figure 3 shows representative Feynman diagrams of the QCD-induced production. The associated production of a ${\text {Z}}$ boson and a single top quark, referred to as ${\text {t}}{\text {Z}}{\text {q}}$ production, is also an important background contribution. Additional background contributions arise from the $\hbox {t}{\bar{\hbox {t}}}$, ${\text {t}}{\text {W}}$, $\hbox {t}{\bar{\hbox {t}}} {\text {W}}$, $\hbox {t}{\bar{\hbox {t}}} {\text {Z}}$, $\hbox {t}{\bar{\hbox {t}}} \gamma$, triple vector boson ($\text {V} \text {V} \text {V}$, $\text {V} ={\text {W}}$, ${\text {Z}}$), and double parton scattering processes.

Multiple Monte Carlo (MC) event generators are used to simulate the signal and background contributions. The signal and background processes are produced with on-shell particles. Three sets of simulated events for each process are needed to match the data taking conditions in the three years. The charged Higgs boson signal samples are simulated using MadGraph 5_amc@nlo 2.4.2 [33, 34] at leading order (LO) accuracy. The predicted signal cross sections are taken at next-to-next-to-LO (NNLO) accuracy from the GM model [21].

The SM EW ${\text {W}}^\pm {\text {W}}^\pm$ and ${\text {W}}{\text {Z}}$ processes, where both bosons decay leptonically, are simulated using MadGraph 5_amc@nlo at LO accuracy with six EW ($\mathcal {O}(\alpha ^6)$) and zero QCD vertices. The same generator is also used to simulate the QCD-induced ${\text {W}}^\pm {\text {W}}^\pm$ process with four EW and two QCD vertices. Contributions with an initial-state ${\text {b}}$ quark are excluded from the EW ${\text {W}}{\text {Z}}$ simulation because they are considered part of the ${\text {t}}{\text {Z}}{\text {q}}$ background process. Triboson processes, where the ${\text {W}}{\text {Z}}$ boson pair is accompanied by a third vector boson that decays into jets, are included in the EW ${\text {W}}{\text {Z}}$ simulation. The QCD-induced ${\text {W}}{\text {Z}}$ process is simulated at LO with up to three additional partons in the matrix element calculations using the MadGraph 5_amc@nlo generator with at least one QCD vertex at tree level. The different jet multiplicities are merged using the MLM scheme [35] to match matrix element and parton shower jets, and the inclusive contribution is normalized to NNLO predictions [36]. The interference between the EW and QCD diagrams is also accounted for with MadGraph 5_amc@nlo.

A complete set of NLO QCD and EW corrections for the leptonic ${\text {W}}^\pm {\text {W}}^\pm$ scattering process has been computed [37, 38] and they reduce the LO cross section of the EW ${\text {W}}^\pm {\text {W}}^\pm$ process by 10–15%, with the correction increasing in magnitude with increasing dilepton and dijet invariant masses. Similarly, the NLO QCD and EW corrections for the leptonic ${\text {W}}{\text {Z}}$ scattering process have been computed at the orders of $\mathcal {O}(\alpha _{S}\alpha ^6)$ and $\mathcal {O}(\alpha ^7)$ [39], reducing the cross sections for the EW ${\text {W}}{\text {Z}}$ process by 10%. The SM EW ${\text {W}}^\pm {\text {W}}^\pm$ and ${\text {W}}{\text {Z}}$ processes are normalized by applying these $\mathcal {O}(\alpha _{S}\alpha ^6)$ and $\mathcal {O}(\alpha ^7)$ corrections to MadGraph 5_amc@nlo LO cross sections.

The ${\textsc {powheg}}$ v2 [40–44] generator is used to simulate the $\hbox {t}{\bar{\hbox {t}}}$, ${\text {t}}{\text {W}}$, ${\text {Z}}{\text {Z}}$, and ${\text {W}}^{\pm }{\text {W}}^{\mp }$ processes at NLO accuracy in QCD. Production of $\hbox {t}{\bar{\hbox {t}}} {\text {W}}$, $\hbox {t}{\bar{\hbox {t}}} {\text {Z}}$, $\hbox {t}{\bar{\hbox {t}}} \gamma$, and $\text {V} \text {V} \text {V}$ events is simulated at NLO accuracy in QCD using the ${\textsc {MadGraph}} {}5\_a{\textsc {mc@nlo}}$ 2.2.2 (2.4.2) generator for the 2016 (2017 and 2018) samples. The ${\text {t}}{\text {Z}}{\text {q}}$ process is simulated in the four-flavor scheme using ${\textsc {MadGraph}} {}5\_a{\textsc {mc@nlo}}$ 2.3.3 at next-to-LO (NLO). Events in which two hard parton-parton interactions occur within a single ${\text {p}}{\text {p}}$ collision, referred to as double parton scattering ${\text {W}}^\pm {\text {W}}^\pm$ production, are generated at LO using ${\textsc {pythia}}$ 8.226 (8.230) [45] for the 2016 (2017 and 2018) samples.

The NNPDF 2.3 LO [46] (NNPDF 3.1 NNLO [47]) PDFs are used for generating 2016 (2017 and 2018) signal samples. The NNPDF 3.0- NLO [48] (NNPDF 3.1 NNLO) PDFs are used for generating all 2016 (2017 and 2018) background samples. For all processes, the parton showering and hadronization are simulated using ${\textsc {pythia}}$ 8.226 (8.230) for 2016 (2017 and 2018). The modeling of the underlying event is done using the CUETP8M1 [49, 50] (CP5 [51]) tune for simulated samples corresponding to the 2016 (2017 and 2018) data.

All MC generated events are processed through a simulation of the CMS detector based on Geant4  [52] and are reconstructed with the same algorithms used for data. The simulated samples include additional interactions in the same and neighboring bunch crossings, referred to as pileup. The additional inelastic events are generated using ${\textsc {pythia}}$ with the same underlying event tune as the main interaction and superimposed on the hard-scattering events. The distribution of the number of pileup interactions in the simulation is adjusted to match the one observed in the data. The average number of interactions per bunch crossing was 23 (32) in 2016 (2017 and 2018) corresponding to an inelastic ${\text {p}}{\text {p}}$ cross-section of 69.2$\,{\text {mb}}$.

## Event reconstruction

The primary vertex (PV) is defined as the vertex with the largest value of summed physics-object $p_{{\mathrm {T}}} ^2$. The physics objects are the jets, clustered using the jet finding algorithm [53, 54] with the tracks assigned to candidate vertices as inputs, and the associated missing transverse momentum, taken as the negative vector sum of the $p_{{\mathrm {T}}}$ of those jets.

The CMS particle-flow (PF) algorithm [55] is used to combine the information from the tracker, calorimeters, and muon systems to reconstruct and identify charged and neutral hadrons, photons, muons, and electrons (PF candidates). The missing transverse momentum vector ${\vec {p}}_{{\mathrm {T}}}^{{\text {miss}}}$ is defined as the projection onto the plane perpendicular to the beam axis of the negative vector momentum sum of all reconstructed PF candidates in an event. Its magnitude is referred to as $p_{{\mathrm {T}}} ^{\text {miss}}$.

Jets are reconstructed by clustering PF candidates using the anti-$k_{{\mathrm {T}}}$ algorithm [53] with a distance parameter of 0.4. Additional proton–proton interactions within the same or nearby bunch crossings can contribute additional tracks and calorimetric energy depositions, increasing the apparent jet momentum. To mitigate this effect, tracks identified to be originating from pileup vertices are discarded and an offset correction is applied to correct for remaining contributions [56]. Jet energy corrections are derived from simulation studies so that the average measured energy of jets becomes identical to that of particle level jets. In situ measurements of the momentum balance in dijet, photon+jet, Z+jet, and multijet events are used to determine any residual differences between the jet energy scale in data and in simulation, and appropriate corrections are made [57]. Corrections to jet energies to account for the detector response are propagated to $p_{{\mathrm {T}}} ^{\text {miss}}$ [58]. Jets with transverse momentum $p_{{\mathrm {T}}} >30\,{\text {GeV}}$ and $|\eta |<4.7$ are included in the analysis.

Events with at least one jet with $p_{{\mathrm {T}}} >20\,{\text {GeV}}$ and $|\eta |<2.4$ that is consistent with the fragmentation of a bottom quark are rejected to reduce the number of top quark background events. The DeepCSV ${\text {b}}$tagging algorithm [59] is used for this selection. For the chosen working point, the efficiency of the algorithm to select ${\text {b}}$quark jets is about 72% and the rate for incorrectly tagging jets originating from the hadronization of gluons or ${\text {u}}$, ${\text {d}}$, ${\text {s}}$ quarks is about 1%. The rate for incorrectly tagging jets originating from the hadronization of $\text {c}$ quarks is about 10%.

Events with at least one reconstructed hadronic decay of a $\tau$ lepton, denoted as ${\uptau }_{\mathrm {h}}$, with $p_{{\mathrm {T}}} >18\,{\text {GeV}}$ and $|\eta |<2.3$, are rejected to reduce the contribution of diboson processes with ${\uptau }_{\mathrm {h}}$ decays. The ${\uptau }_{\mathrm {h}}$ decays are reconstructed using the hadrons-plus-strips algorithm [60].

Electrons and muons are reconstructed by associating a track reconstructed in the tracking detectors with either a cluster of energy deposits in the ECAL [61, 62] or a track in the muon system [63]. Electrons (muons) must pass loose identification criteria with $p_{{\mathrm {T}}} >10\,{\text {GeV}}$ and $|\eta |<2.5$ (2.4) to be selected for the analysis. At the final stage of the lepton selection, tight working points, following the definitions provided in Refs. [61–63], are chosen for the identification criteria, including requirements on the impact parameter of the candidates with respect to the PV and their isolation with respect to other particles in the event [64]. For electrons, the background contribution arising from charge misidentification is not negligible. The sign mismeasurement is evaluated using three observables that measure the electron curvature applying different methods as discussed in Ref. [61]. Requiring all three charge evaluations to agree reduces this background contribution by a factor of four (six) with an efficiency of about 97 (90)% in the barrel (endcap) region. The sign mismeasurement is negligible for muons [65, 66].

## Event selection

Collision events are collected using single-electron and single-muon triggers that require the presence of an isolated lepton with $p_{{\mathrm {T}}} >27$ and 24$\,{\text {GeV}}$, respectively [67]. In addition, a set of dilepton triggers with lower $p_{{\mathrm {T}}}$ thresholds is used, ensuring a trigger efficiency above 99% for events that satisfy the subsequent offline selection [67].

Several selection requirements are used to isolate the ${\text {W}}^\pm {\text {W}}^\pm$ and ${\text {W}}{\text {Z}}$ topologies defining the signal regions (SRs), while reducing the contributions from background processes [26]. Candidate events must contain exactly two isolated same-sign charged leptons or exactly three isolated charged leptons with $p_{{\mathrm {T}}} >10\,{\text {GeV}}$, and at least two jets with $|\eta |<4.7$ and the leading jet $p_{{\mathrm {T}}} ^{\mathrm {j}}>50\,{\text {GeV}}$. To exclude the selected electrons and muons from the jet sample, the jets are required to be separated from the identified leptons by $\Delta R = \sqrt{\smash [b]{(\Delta \eta )^{2} + (\Delta \phi )^{2}}} > 0.4$, where $\phi$ is the azimuthal angle in radians.

For the ${\text {W}}{\text {Z}}$ candidate events, one of the oppositely charged same-flavor leptons from the ${\text {Z}}$ boson candidate is required to have $p_{{\mathrm {T}}} >25\,{\text {GeV}}$ and the other $p_{{\mathrm {T}}} >10\,{\text {GeV}}$ with the invariant mass of the dilepton pair $\mathrm {m}_{\ell \ell }$ satisfying $|\mathrm {m}_{\ell \ell }- m_{{\text {Z}}} |<15\,{\text {GeV}}$. For candidate events with three same-flavor leptons, the oppositely charged lepton pair with the invariant mass closest to the world-average ${\text {Z}}$ boson mass $m_{{\text {Z}}}$ [68] is selected as the ${\text {Z}}$ boson candidate. The third lepton associated with the ${\text {W}}$ boson is required to have $p_{{\mathrm {T}}} >20\,{\text {GeV}}$. In addition, the trilepton invariant mass $m_{\ell \ell \ell }$ is required to exceed 100$\,{\text {GeV}}$ to exclude a region where production of ${\text {Z}}$ bosons with final-state photon radiation is expected to contribute.

One of the leptons in the same-sign ${\text {W}}^\pm {\text {W}}^\pm$ candidate events is required to have $p_{{\mathrm {T}}} >25\,{\text {GeV}}$ and the other $p_{{\mathrm {T}}} >20\,{\text {GeV}}$. The value of $\mathrm {m}_{\ell \ell }$ must be greater than 20$\,{\text {GeV}}$. Candidate events in the dielectron final state with $|\mathrm {m}_{\ell \ell }-m_{{\text {Z}}} |<15\,{\text {GeV}}$ are rejected to reduce the number of ${\text {Z}}$ boson background events where the sign of one of the electron candidates is misidentified.

The VBF topology is targeted by requiring the two highest $p_{{\mathrm {T}}}$ jets to have a mass $m_{\mathrm {j}\mathrm {j}} >500\,{\text {GeV}}$ and a pseudorapidity separation $|\Delta \eta _{\mathrm {j}\mathrm {j}} | >2.5$. The ${\text {W}}$ and ${\text {Z}}$ bosons in the VBF topologies are mostly produced in the central rapidity region with respect to the two selected jets. The candidate ${\text {W}}^\pm {\text {W}}^\pm$ (${\text {W}}{\text {Z}}$) events are required to satisfy $\mathrm {max}(\mathrm {z}_{\ell }^{*})<0.75 (1.0)$, where $\mathrm {z}_{\ell }^{*}=|\eta ^{\ell } - (\eta ^{\mathrm {j}_{1}} + \eta ^{\mathrm {j}_{2}})/2 |/|\Delta \eta _{\mathrm {j}\mathrm {j}} |$ is the Zeppenfeld variable [69] for one of the selected leptons. Here $\eta ^{\ell }$ is the pseudorapidity of the lepton, and $\eta ^{\mathrm {j}_{1}}$ and $\eta ^{\mathrm {j}_{2}}$ are the pseudorapidities of the two candidates VBF jets.

The $p_{{\mathrm {T}}} ^{\text {miss}}$ is required to exceed 30$\,{\text {GeV}}$ for both SRs. The selection requirements used to define the same-sign ${\text {W}}^\pm {\text {W}}^\pm$ and ${\text {W}}{\text {Z}}$ SRs are summarized in Table 1.

**Table 1:** Summary of the selection requirements defining the ${\text {W}}^\pm {\text {W}}^\pm$ and ${\text {W}}{\text {Z}}$ SRs. The looser lepton $p_{{\mathrm {T}}}$ requirement in the ${\text {W}}{\text {Z}}$ selection refers to the trailing lepton from the ${\text {Z}}$ boson decays. The $|\mathrm {m}_{\ell \ell }- m_{{\text {Z}}} |$ requirement is applied only to the dielectron final state in the ${\text {W}}^\pm {\text {W}}^\pm$ SR

Variable	${\text {W}}^\pm {\text {W}}^\pm$	${\text {W}}{\text {Z}}$
Leptons	2 leptons, $p_{{\mathrm {T}}} >25/20\,{\text {GeV}}$	3 leptons, $p_{{\mathrm {T}}} >25/10/20\,{\text {GeV}}$
$p_{{\mathrm {T}}} ^\mathrm {j}$	>50/30$\,{\text {GeV}}$	>50/30$\,{\text {GeV}}$
$|\mathrm {m}_{\ell \ell }- m_{{\text {Z}}} |$	>15$\,{\text {GeV}}$ (${\text {e}}{\text {e}}$)	<15$\,{\text {GeV}}$
$\mathrm {m}_{\ell \ell }$	>20$\,{\text {GeV}}$	–
$m_{\ell \ell \ell }$	–	>100$\,{\text {GeV}}$
$p_{{\mathrm {T}}} ^{\text {miss}}$	>30$\,{\text {GeV}}$	>30$\,{\text {GeV}}$
${\text {b}}$jet veto	Required	Required
${\uptau }_{\mathrm {h}}$ veto	Required	Required
$\max (z_{\ell }^{*})$	<0.75	<1.0
$m_{\mathrm {j}\mathrm {j}}$	>500$\,{\text {GeV}}$	>500$\,{\text {GeV}}$
$|\Delta \eta _{\mathrm {j}\mathrm {j}} |$	>2.5	>2.5

## Background estimation

A combination of methods based on simulation and on control regions (CRs) in data is used to estimate background contributions. By inverting some of the requirements in Table 1 we select background-enriched CRs. Uncertainties related to the theoretical and experimental predictions are estimated as described in Sect. 8.

The nonprompt lepton backgrounds originating from leptonic decays of heavy quarks, hadrons misidentified as leptons, and electrons from photon conversions are suppressed by the identification and isolation requirements imposed on leptons. The remaining contribution from the nonprompt lepton background is dominant in the ${\text {W}}^\pm {\text {W}}^\pm$ SR and is estimated directly from data following the technique described in Ref. [70], using events selected by the final selection criteria, except for one of the leptons, which is requested to pass a looser criterion having failed the nominal selection. The yield in this sample is extrapolated to the signal region using the efficiencies for such loosely identified leptons to pass the standard lepton selection criteria. This efficiency is calculated in a sample of events dominated by dijet production. An uncertainty of 20% is assigned for the nonprompt lepton background normalization to include possible differences in the composition of jets between the data sample used to derive these efficiencies and the data samples in the ${\text {W}}^\pm {\text {W}}^\pm$ and ${\text {W}}{\text {Z}}$ SRs [64].

**Fig. 4:**
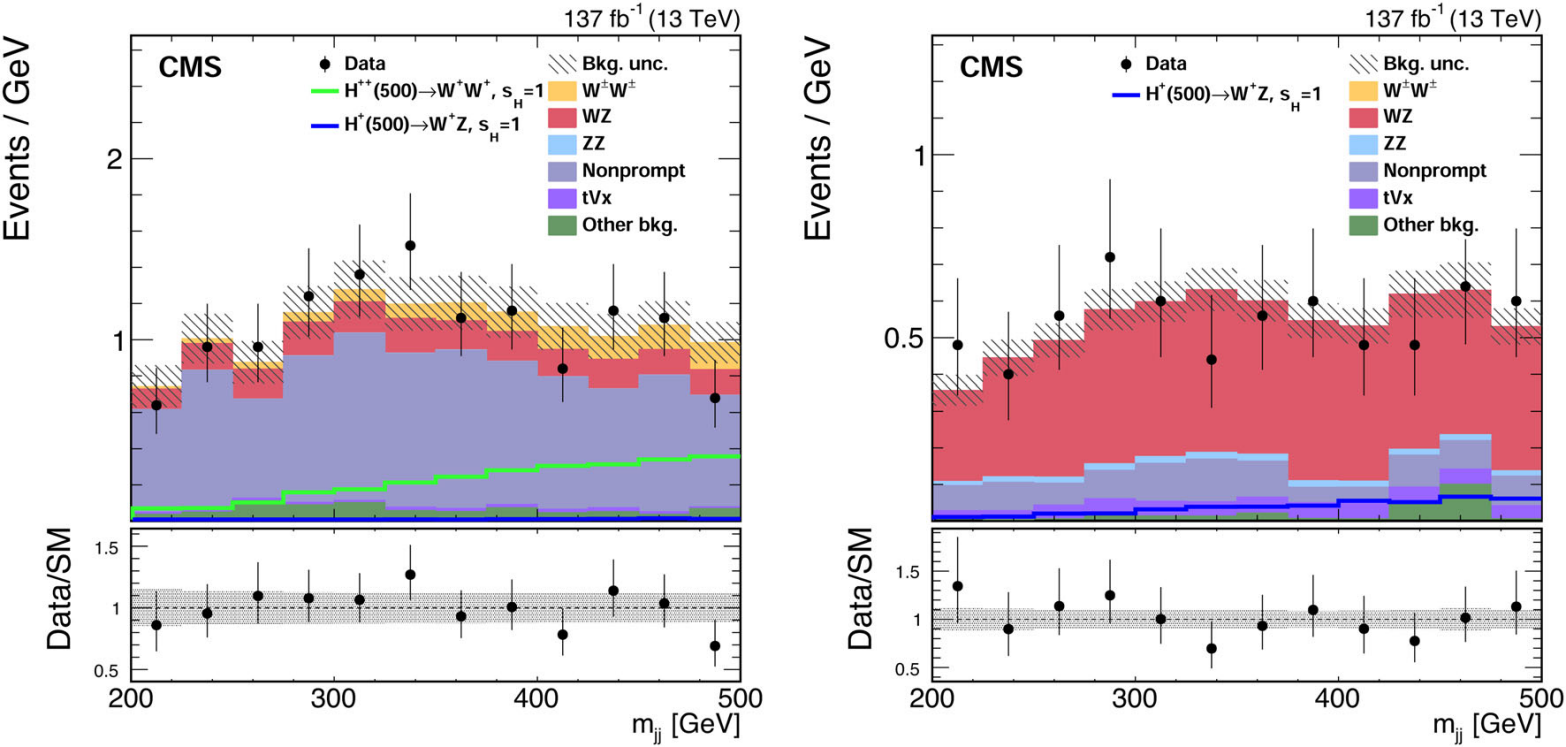
The $m_{\mathrm {j}\mathrm {j}}$ distributions after requiring the same selection as for the ${\text {W}}{\text {W}}$ (upper) and ${\text {W}}{\text {Z}}$ (lower) SRs, but with a requirement of $200<m_{\mathrm {j}\mathrm {j}} <500\,{\text {GeV}}$. The predicted yields are shown with their best fit normalizations from the simultaneous fit (described in Sect. 7) for the background-only hypothesis i.e., assuming no contributions from the ${\text {H}} ^{\pm }$ and ${\text {H}} ^{\pm \pm }$ processes. Vertical bars on data points represent the statistical uncertainty in the data. The histograms for ${\text {t}}\text {V} \mathrm {x}$ backgrounds include the contributions from $\hbox {t}{\bar{\hbox {t}}} \text {V}$ and ${\text {t}}{\text {Z}}{\text {q}}$ processes. The histograms for other backgrounds include the contributions from double parton scattering, $\text {V} \text {V} \text {V}$, and from oppositely charged dilepton final states from $\hbox {t}{\bar{\hbox {t}}}$, ${\text {t}}{\text {W}}$, ${\text {W}}^{+}{\text {W}}^{-}$, and Drell–Yan processes. The overflow is included in the last bin. The lower panels show the ratio of the number of events observed in data to that of the total SM prediction. The hatched gray bands represent the uncertainties in the predicted yields. The solid lines show the signal predictions for values of $s_{{\text {H}}}=1.0$ and $m_{{\text {H}} _{5}}=500\,{\text {GeV}}$ in the GM model

The background contribution from the electron sign mismeasurement is estimated from the simulation by applying a data-to-simulation efficiency correction due to electrons with sign mismeasurement. These corrections are determined using ${\text {Z}}\rightarrow {\text {e}}{\text {e}}$ events in the ${\text {Z}}$ boson peak region that were recorded with independent triggers. These corrections amount to 40% for data collected in 2017 and 2018, while they are negligible for 2016 data. The electron sign mismeasurement rate is about 0.01 (0.3)% in the barrel (endcap) region [61, 62].

Three CRs are used to select nonprompt lepton, ${\text {t}}{\text {Z}}{\text {q}}$, and ${\text {Z}}{\text {Z}}$ background-enriched events to further estimate the normalization of these background processes from data. The nonprompt lepton CR is defined by requiring the same selection as for the ${\text {W}}^\pm {\text {W}}^\pm$ SR, but with the ${\text {b}}$jet veto requirement inverted. The selected events are enriched in the nonprompt lepton background coming mostly from semileptonic $\hbox {t}{\bar{\hbox {t}}}$ events. Similarly, the ${\text {t}}{\text {Z}}{\text {q}}$ CR is defined by requiring the same selection as for the ${\text {W}}{\text {Z}}$ SR, but with the ${\text {b}}$jet veto requirement inverted. The selected events are dominated by the ${\text {t}}{\text {Z}}{\text {q}}$ background process. Finally, the ${\text {Z}}{\text {Z}}$ CR selects events with two opposite-sign same-flavor lepton pairs with the same VBS-like requirements. The three CRs are used together with the SRs to constrain the normalization of the nonprompt lepton, ${\text {t}}{\text {Z}}{\text {q}}$, and ${\text {Z}}{\text {Z}}$ background processes from data. All other background processes are estimated from simulation after applying corrections to account for the small differences between data and simulation. The shapes of the ${\text {t}}{\text {Z}}{\text {q}}$ and ${\text {Z}}{\text {Z}}$ background processes are estimated from simulation as well.

The prediction for the QCD ${\text {W}}{\text {Z}}$ background process is validated in a CR defined by requiring the same selection as for the ${\text {W}}{\text {Z}}$ SR, but with a requirement of $200<m_{\mathrm {j}\mathrm {j}} <500\,{\text {GeV}}$. The predicted yields are shown with their best fit normalizations from the simultaneous fit (described in Sect. 7) for the background-only hypothesis i.e., assuming no contributions from the ${\text {H}} ^{\pm }$ and ${\text {H}} ^{\pm \pm }$ processes. Good agreement between the data and post-fit predicted yields is observed in this CR as can be seen in Fig. 4.

## Signal extraction

A binned maximum-likelihood fit is performed using the ${\text {W}}^\pm {\text {W}}^\pm$ and ${\text {W}}{\text {Z}}$ SRs, and the nonprompt lepton, ${\text {t}}{\text {Z}}{\text {q}}$, and ${\text {Z}}{\text {Z}}$ CRs to discriminate between the signal and the remaining backgrounds. Signal contributions with electrons and muons produced in the decay of a $\tau$ lepton are included. The normalization factors for the ${\text {t}}{\text {Z}}{\text {q}}$ and ${\text {Z}}{\text {Z}}$ background processes, affecting both the SRs and CRs, are included as free parameters in the maximum-likelihood fit together with the signal strength. The SM ${\text {W}}^\pm {\text {W}}^\pm$ (${\text {W}}{\text {Z}}$) contribution is obtained from the sum of the EW ${\text {W}}^\pm {\text {W}}^\pm$ (${\text {W}}{\text {Z}}$), QCD ${\text {W}}^\pm {\text {W}}^\pm$ (${\text {W}}{\text {Z}}$), and the interference contributions according to the SM predictions [26] and allowed to vary within the uncertainties.

The diboson transverse mass ($m_{\text {T}} ^{\text {V} \text {V}}$) is constructed from the four-momentum of the selected charged leptons and the ${\vec {p}}_{{\mathrm {T}}}^{{\text {miss}}}$. The four-momentum of the neutrino system is defined using the ${\vec {p}}_{{\mathrm {T}}}^{{\text {miss}}}$, assuming that the values of the longitudinal component of the momentum and the mass are zero. The value of $m_{\text {T}} ^{\text {V} \text {V}}$, defined as

1 $$\begin{aligned} m_{\text {T}} ^{\text {V} \text {V}} = \sqrt{{{\biggl (\sum \nolimits _i E_{i}\biggr )^2-\biggl (\sum \nolimits _i p_{z,i}\biggr )^2}}}, \end{aligned}$$

where $E_{i}$ and $p_{z,i}$ are the energies and longitudinal components of the momenta of the leptons and neutrino system from the decay of the gauge bosons in the event, is effective in discriminating between the resonant signal and nonresonant background processes. The value of $m_{\mathrm {j}\mathrm {j}}$ is effective in discriminating between all non-VBS processes and the signal (plus EW $\text {V} \text {V}$) processes because VBF and VBS topologies typically exhibit large values for the dijet mass. A two-dimensional distribution is used in the fit for the ${\text {W}}^\pm {\text {W}}^\pm$ SR with 8 bins in $m_{\text {T}} ^{\text {V} \text {V}}$ ([0, 250, 350, 450, 550, 650, 850, 1050, $\infty$]$\,{\text {GeV}}$) and 4 bins in $m_{\mathrm {j}\mathrm {j}}$ ([500, 800, 1200, 1800, $\infty$]$\,{\text {GeV}}$). Similarly, a two-dimensional distribution is used in the fit for the ${\text {W}}{\text {Z}}$ SR with 7 bins in $m_{\text {T}} ^{\text {V} \text {V}}$ ([0, 325, 450, 550, 650, 850, 1350, $\infty$]$\,{\text {GeV}}$) and 2 bins in $m_{\mathrm {j}\mathrm {j}}$ ([500, 1500, $\infty$]$\,{\text {GeV}}$). The $m_{\mathrm {j}\mathrm {j}}$ distribution is used for the CRs in the fit with 4 bins ([500, 800, 1200, 1800, $\infty$]$\,{\text {GeV}}$).

A profile likelihood technique is used where systematic uncertainties are represented by nuisance parameters [71]. For each individual bin, a Poisson likelihood term describes the fluctuation of the data around the expected central value, which is given by the sum of the contributions from signal and background processes. The systematic uncertainties are treated as nuisance parameters and are profiled with the shape and normalization of each distribution varying within the respective uncertainties in the fit. The normalization uncertainties are treated as log-normal nuisance parameters. Correlation across bins is taken into account. The uncertainties affecting the shapes of the distributions are modeled in the fit as nuisance parameters with external Gaussian constraints. The dominant nuisance parameters are not significantly constrained by the data, i.e., the normalized nuisance parameter uncertainties are close to unity.

## Systematic uncertainties

Several sources of systematic uncertainty are taken into account in the signal extraction procedure. For each source of uncertainty, the effects on the signal and background distributions are considered to be correlated.

The total Run 2 (2016–2018) integrated luminosity has an uncertainty of 1.8%, the improvement in precision relative to Refs. [22–24] reflecting the (uncorrelated) time evolution of some systematic effects.

The simulation of pileup events assumes an inelastic ${\text {p}}{\text {p}}$ cross section of 69.2$\,{\text {mb}}$, with an associated uncertainty of 5% [72], which has an impact on the expected signal and background yields of about 1%.

Discrepancies in the lepton reconstruction and identification efficiencies between data and simulation are corrected by applying scale factors to all simulation samples. These scale factors, which depend on the $p_{{\mathrm {T}}}$ and $\eta$ for both electrons and muons, are determined using ${\text {Z}}\rightarrow \ell \ell$ events in the ${\text {Z}}$ boson peak region that were recorded with independent triggers [61, 63, 73]. The uncertainty in the determination of the trigger efficiency leads to an uncertainty smaller than 1% in the expected signal yield. The trigger efficiency in the simulation is corrected to account for the effect of a gradual time shifts in the forward region in the ECAL endcaps for the 2016 and 2017 data [74]. The uncertainty in this correction is included in the trigger efficiency uncertainty. The lepton momentum scale uncertainty is computed by varying the lepton momenta in simulation with their uncertainties, and repeating the analysis selection. The resulting uncertainties in the yields are $\approx$1% for both electrons and muons. These uncertainties are assumed to be correlated across the three data sets.

The uncertainty in the calibration of the jet energy scale (JES) directly affects the acceptance of the jet multiplicity requirement and the $p_{{\mathrm {T}}} ^{\text {miss}}$ measurement. These effects are estimated by shifting the JES in the simulated samples up and down by one standard deviation. The uncertainty in the jet energy resolution (JER) smearing applied to simulated samples to match the $p_{{\mathrm {T}}}$ resolution measured in data causes both a change in the normalization and in the shape of the distributions. The overall uncertainty in the JES and JER is 2–5%, depending on $p_{{\mathrm {T}}}$ and $\eta$ [57, 75], and its impact on the expected signal and background yields is about 3%.

**Table 2:** Summary of the impact of the systematic uncertainties on the extracted signal strength; for the case of a background-only simulated data set, i.e., assuming no contributions from the ${\text {H}} ^{\pm }$ and ${\text {H}} ^{\pm \pm }$ processes, and including a charged Higgs boson signal for values of $s_{{\text {H}}}=1.0$ and $m_{{\text {H}} _{5}}=500\,{\text {GeV}}$ in the GM model. The impacts shown result from a fit to two simulated samples: background-only (first column, expected $\mu = 0$) and signal-plus-background (second column, expected $\mu = 1$)

Source of uncertainty	$\Delta \mu$	$\Delta \mu$
	Background-only	$s_{{\text {H}}}=1.0$ and $m_{{\text {H}} _{5}}=500\,{\text {GeV}}$
Integrated luminosity	0.002	0.019
Pileup	0.001	0.001
Lepton measurement	0.003	0.033
Trigger	0.001	0.007
JES and JER	0.003	0.006
${\text {b}}$tagging	0.001	0.006
Nonprompt rate	0.002	0.002
${\text {W}}^\pm {\text {W}}^\pm /{\text {W}}{\text {Z}}$ rate	0.014	0.015
Other prompt background rate	0.002	0.015
Signal rate	–	0.064
Limited sample size	0.005	0.005
Total systematic uncertainty	0.016	0.078
Statistical uncertainty	0.021	0.044
Total uncertainty	0.027	0.090

**Fig. 5:**
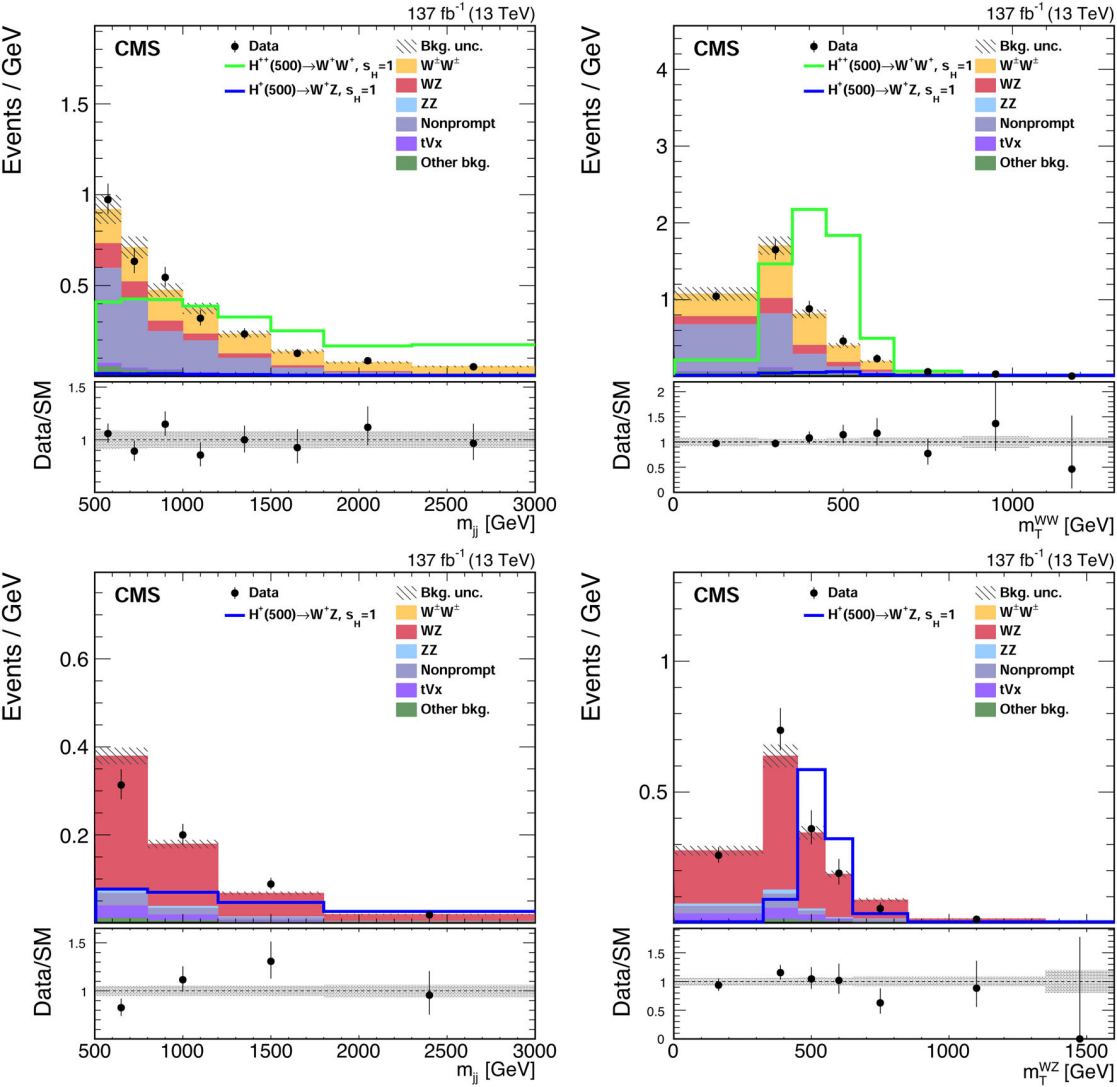
The $m_{\mathrm {j}\mathrm {j}}$ (upper left) and $m_{\text {T}} ^{{\text {W}}{\text {W}}}$ (upper right) distributions in the ${\text {W}}{\text {W}}$ SR, and the $m_{\mathrm {j}\mathrm {j}}$ (lower left) and $m_{\text {T}} ^{{\text {W}}{\text {Z}}}$ (lower right) distributions in the ${\text {W}}{\text {Z}}$ SR for signal, backgrounds, and data. The predicted yields are shown with their best fit normalizations from the simultaneous fit for the background-only hypothesis, i.e., assuming no contributions from the ${\text {H}} ^{\pm }$ and ${\text {H}} ^{\pm \pm }$ processes. Vertical bars on data points represent the statistical uncertainty in the data. The histograms for ${\text {t}}\text {V} \mathrm {x}$ backgrounds include the contributions from $\hbox {t}{\bar{\hbox {t}}} \text {V}$ and ${\text {t}}{\text {Z}}{\text {q}}$ processes. The histograms for other backgrounds include the contributions from double parton scattering, $\text {V} \text {V} \text {V}$, and from oppositely charged dilepton final states from $\hbox {t}{\bar{\hbox {t}}}$, ${\text {t}}{\text {W}}$, ${\text {W}}^{+}{\text {W}}^{-}$, and Drell–Yan processes. The overflow is included in the last bin. The lower panels show the ratio of the number of events observed in data to that of the total SM prediction. The hatched gray bands represent the uncertainties in the predicted yields. The solid lines show the signal predictions for values of $s_{{\text {H}}}=1.0$ and $m_{{\text {H}} _{5}}=500\,{\text {GeV}}$ in the GM model

The ${\text {b}}$ tagging efficiency in the simulation is corrected using scale factors determined from data [59]. These values are estimated separately for correctly and incorrectly tagged jets. Each set of values results in uncertainties in the ${\text {b}}$ tagging efficiency of about 1–4% depending on $p_{{\mathrm {T}}}$ and $\eta$, and the impact on the expected signal and background yields is about 1%. The uncertainties in the JER, JES and ${\text {b}}$ tagging are treated as uncorrelated across the three data taking years, since the detector conditions have changed among the three years.

**Table 3:** Expected signal and background yields from various SM processes and observed data events in all regions used in the analysis. The expected background yields are shown with their normalizations from the simultaneous fit for the background-only hypothesis, i.e., assuming no contributions from the ${\text {H}} ^{\pm }$ and ${\text {H}} ^{\pm \pm }$ processes. The expected signal yields are shown for $s_{{\text {H}}}=1.0$ in the GM model. The combination of the statistical and systematic uncertainties is shown

Process	${\text {W}}{\text {W}}$ SR	${\text {W}}{\text {Z}}$ SR	Nonprompt CR	${\text {t}}{\text {Z}}{\text {q}}$ CR	${\text {Z}}{\text {Z}}$ CR
${\text {H}} ^{\pm \pm }(500) \rightarrow {\text {W}}^\pm {\text {W}}^\pm$	666 ± 68	–	48.9 ± 5.1	–	–
${\text {H}} ^{\pm }(500) \rightarrow {\text {W}}{\text {Z}}$	19.2 ± 2.4	107 ± 11	1.7 ± 0.2	8.0 ± 0.9	–
${\text {W}}^\pm {\text {W}}^\pm$	230 ± 16	–	28.2 ± 1.8	–	–
${\text {W}}{\text {Z}}$	67.8 ± 5.8	196 ± 15	10.3 ± 1.0	27.2 ± 2.4	–
${\text {Z}}{\text {Z}}$	0.7 ± 0.2	6.4 ± 2.0	0.1 ± 0.1	1.1 ± 0.3	13.3 ± 4.0
Nonprompt	262 ± 36	22.3 ± 7.7	263 ± 21	8.4 ± 3.1	0.2 ± 0.2
${\text {t}}\text {V} \mathrm {x}$	8.4 ± 1.9	17.7 ± 3.3	28.8 ± 5.6	62 ± 11	0.2 ± 0.1
Other background	31.1 ± 7.3	6.8 ± 1.4	21.1 ± 4.2	2.2 ± 0.4	0.3 ± 0.1
Total background	600 ± 40	249 ± 18	352 ± 22	101 ± 12	14.0 ± 4.0
Data	602	249	352	101	14

The theoretical uncertainties associated with the choice of the renormalization and factorization scales are estimated by varying these scales independently up and down by a factor of two from their nominal values. The envelope of the resulting distributions, excluding the two extreme variations where one scale is varied up and the other one down, is taken as the uncertainty [76, 77]. The variations of the PDF set and $\alpha _{S}$ are used to estimate the corresponding uncertainties in the yields of the signal and background processes, following Refs. [48, 78]. The uncertainty in the yields due to missing higher-order EW corrections in the GM model is estimated to be 7% [21]. These theoretical uncertainties may affect both the estimated signal and background rates. The statistical uncertainties that are associated with the limited number of simulated events and data events used to estimate the nonprompt lepton background are also considered as systematic uncertainties.

**Fig. 6:**
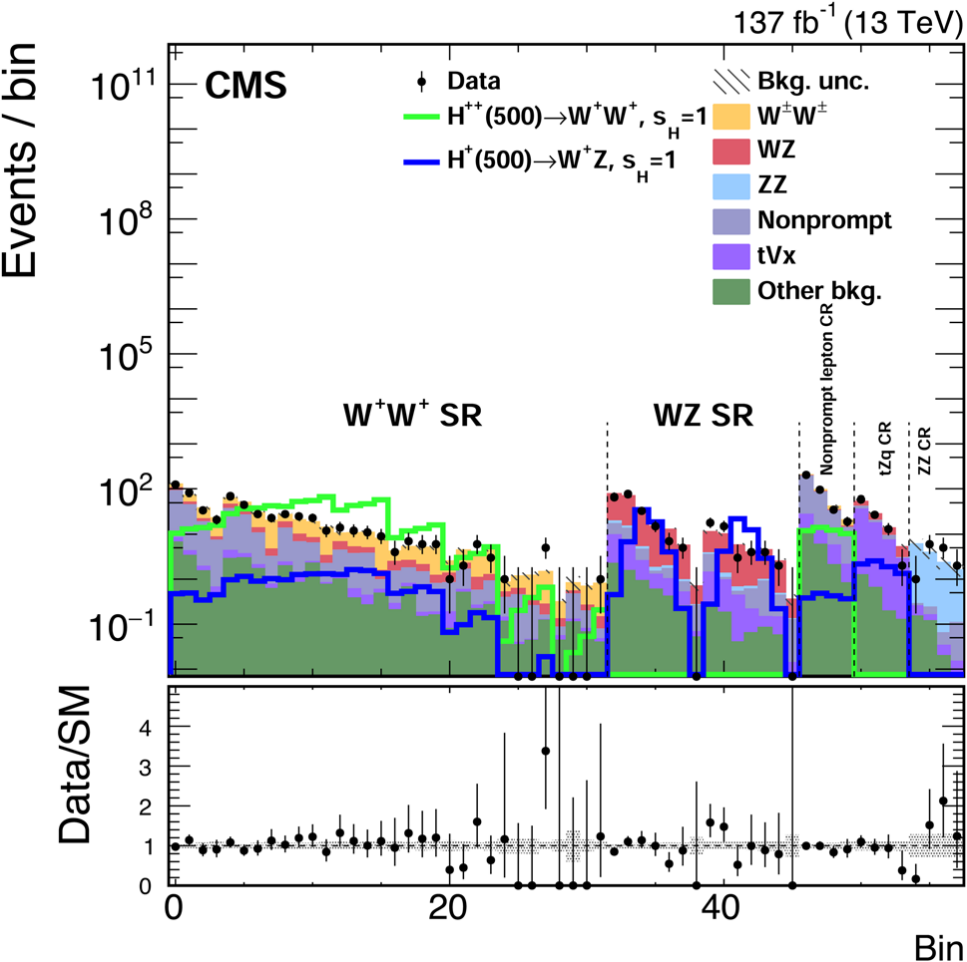
Distributions for signal, backgrounds, and data for the bins used in the simultaneous fit. The bins 1–32 (4$\times$8) show the events in the ${\text {W}}{\text {W}}$ SR ($m_{\mathrm {j}\mathrm {j}} \times m_{\text {T}}$), the bins 33–46 (2$\times$7) show the events in the ${\text {W}}{\text {Z}}$ SR ($m_{\mathrm {j}\mathrm {j}} \times m_{\text {T}}$), the 4 bins 47–50 show the events in the nonprompt lepton CR ($m_{\mathrm {j}\mathrm {j}}$), the 4 bins 51–54 show the events in the ${\text {t}}{\text {Z}}{\text {q}}$ CR ($m_{\mathrm {j}\mathrm {j}}$), and the 4 bins 55–58 show the events in the ${\text {Z}}{\text {Z}}$ CR ($m_{\mathrm {j}\mathrm {j}}$). The predicted yields are shown with their best fit normalizations from the simultaneous fit for the background-only hypothesis, i.e., assuming no contributions from the ${\text {H}} ^{\pm }$ and ${\text {H}} ^{\pm \pm }$ processes. Vertical bars on data points represent the statistical uncertainty in the data. The histograms for ${\text {t}}\text {V} \mathrm {x}$ backgrounds include the contributions from $\hbox {t}{\bar{\hbox {t}}} \text {V}$ and ${\text {t}}{\text {Z}}{\text {q}}$ processes. The histograms for other backgrounds include the contributions from double parton scattering, $\text {V} \text {V} \text {V}$, and from oppositely charged dilepton final states from $\hbox {t}{\bar{\hbox {t}}}$, ${\text {t}}{\text {W}}$, ${\text {W}}^{+}{\text {W}}^{-}$, and Drell–Yan processes. The overflow is included in the last bin in each corresponding region. The lower panels show the ratio of the number of events observed in data to that of the total SM prediction. The hatched gray bands represent the uncertainties in the predicted yields. The solid lines show the signal predictions for values of $s_{{\text {H}}}=1.0$ and $m_{{\text {H}} _{5}}=500\,{\text {GeV}}$ in the GM model

A summary of the impact of the systematic uncertainties on the signal strength, $\mu$, defined as the ratio of the observed charged Higgs signal yield to the expected yield, is shown in Table 2 for the case of a background-only simulated data set, i.e., assuming no contributions from the ${\text {H}} ^{\pm }$ and ${\text {H}} ^{\pm \pm }$ processes. Table 2 also shows systematic uncertainties including a charged Higgs boson signal for values of $s_{{\text {H}}}=1.0$ and $m_{{\text {H}} _{5}}=500\,{\text {GeV}}$ in the GM model. The impacts shown in Table 2 result from a fit to two simulated samples: background-only (first column, expected $\mu = 0$) and signal-plus-background (second column, expected $\mu = 1$). They differ from the impacts in percent on the expected signal and background yields given above, which are estimated before the fit. The total systematic uncertainty is smaller for the background-only simulated data set because the uncertainties partially cancel out between the SRs and the CRs for the background processes.

## Results

**Fig. 7:**
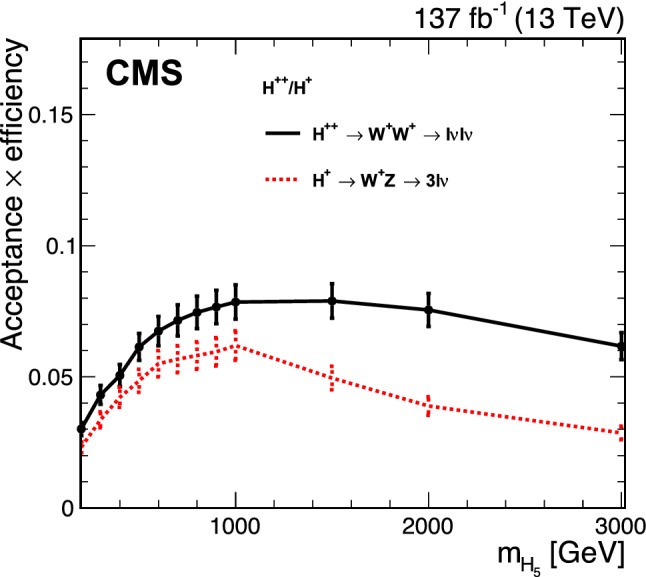
The product of acceptance and selection efficiency within the fiducial region for the VBF ${\text {H}} ^{\pm \pm }\rightarrow {\text {W}}^\pm {\text {W}}^\pm \rightarrow 2\ell 2\nu$ and ${\text {H}} ^{\pm }\rightarrow {\text {W}}{\text {Z}} \rightarrow 3\ell \nu$ processes, as a function of $m_{{\text {H}} _{5}}$. The combination of the statistical and systematic uncertainties is shown. The theoretical uncertainties in the acceptance are also included

The distributions of $m_{\mathrm {j}\mathrm {j}}$ and $m_{\text {T}} ^{\text {V} \text {V}}$ in the ${\text {W}}{\text {W}}$ and ${\text {W}}{\text {Z}}$ SRs are shown in Fig. 5. The $m_{\mathrm {j}\mathrm {j}}$ distributions in the ${\text {W}}{\text {W}}$ and ${\text {W}}{\text {Z}}$ SRs are shown with finer binning compared to the binning used in the two-dimensional distribution in the fit. Distributions for signal, backgrounds, and data for the bins used in the simultaneous fit are shown in Fig. 6. The data yields, together with the background expectations with the best fit normalizations for the background-only hypothesis, i.e., assuming no contributions from the ${\text {H}} ^{\pm }$ and ${\text {H}} ^{\pm \pm }$ processes, are shown in Table 3. The product of kinematic acceptance and selection efficiency within the fiducial region for the ${\text {H}} ^{\pm \pm }\rightarrow {\text {W}}^\pm {\text {W}}^\pm \rightarrow 2\ell 2\nu$ and ${\text {H}} ^{\pm }\rightarrow {\text {W}}{\text {Z}} \rightarrow 3\ell \nu$ processes, as a function of $m_{{\text {H}} _{5}}$, is shown in Fig. 7. The drop of selection efficiency for the ${\text {H}} ^{\pm }\rightarrow {\text {W}}{\text {Z}} \rightarrow 3\ell \nu$ process for masses above $1000\,{\text {GeV}}$ is coming from the lepton isolation requirement as the leptons from high-momentum ${\text {Z}}$ boson decay are produced with a small angular separation.

**Fig. 8:**
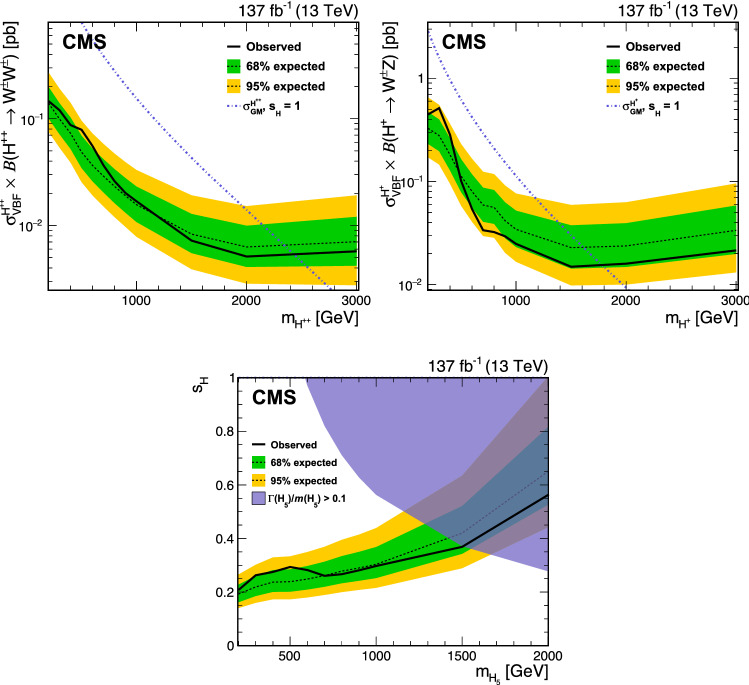
Expected and exclusion limits at 95% CL for $\sigma _{\mathrm {VBF}}({\text {H}} ^{\pm \pm }) \, {\mathcal {B}}({\text {H}} ^{\pm \pm }\rightarrow {\text {W}}^\pm {\text {W}}^\pm )$ as functions of $m_{{\text {H}} ^{\pm \pm }}$ (upper left), for $\sigma _{\mathrm {VBF}}({\text {H}} ^{\pm }) \, {\mathcal {B}}({\text {H}} ^{\pm }\rightarrow {\text {W}}{\text {Z}})$ as functions of $m_{{\text {H}} ^{\pm }}$ (upper right), and for $s_{{\text {H}}}$ as functions of $m_{{\text {H}} _{5}}$ in the GM model (lower). The contribution of the ${\text {H}} ^{\pm }$ (${\text {H}} ^{\pm \pm }$) boson signal is set to zero for the derivation of the exclusion limits on the $\sigma _{\mathrm {VBF}}({\text {H}} ^{\pm \pm }) \, {\mathcal {B}}({\text {H}} ^{\pm \pm }\rightarrow {\text {W}}^\pm {\text {W}}^\pm )$ ($\sigma _{\mathrm {VBF}}({\text {H}} ^{\pm }) \, {\mathcal {B}}({\text {H}} ^{\pm }\rightarrow {\text {W}}{\text {Z}})$). The exclusion limits for $s_{{\text {H}}}$ are shown up to $m_{{\text {H}} _{5}}=2000\,{\text {GeV}}$, given the low sensitivity in the GM model for values above that mass. Values above the curves are excluded

No significant excess of events above the expectation from the SM background predictions is found. The 95% ${\text {CL}}$ upper limits on the charged Higgs production cross sections are calculated using the modified frequentist approach with the $\text {CL}_\text {s}$ criterion [79, 80] and asymptotic method for the test statistic [71, 81].

Constraints on resonant charged Higgs boson production are derived. The exclusion limits on the product of the doubly charged Higgs boson cross section and branching fraction $\sigma _{\mathrm {VBF}}({\text {H}} ^{\pm \pm }) \, {\mathcal {B}}({\text {H}} ^{\pm \pm }\rightarrow {\text {W}}^\pm {\text {W}}^\pm )$ at 95% ${\text {CL}}$as a function of $m_{{\text {H}} ^{\pm \pm }}$ are shown in Fig. 8 (upper left). The exclusion limits on the product of the charged Higgs boson cross section and branching fraction $\sigma _{\mathrm {VBF}}({\text {H}} ^{\pm }) \, {\mathcal {B}}({\text {H}} ^{\pm }\rightarrow {\text {W}}{\text {Z}})$ at 95% ${\text {CL}}$as a function of $m_{{\text {H}} ^{\pm }}$ are shown in Fig. 8 (upper right). The contributions of the ${\text {H}} ^{\pm }$ and ${\text {H}} ^{\pm \pm }$ boson signals are set to zero for the derivation of the individual exclusion limits on $\sigma _{\mathrm {VBF}}({\text {H}} ^{\pm \pm }) \, {\mathcal {B}}({\text {H}} ^{\pm \pm }\rightarrow {\text {W}}^\pm {\text {W}}^\pm )$ and $\sigma _{\mathrm {VBF}}({\text {H}} ^{\pm }) \, {\mathcal {B}}({\text {H}} ^{\pm }\rightarrow {\text {W}}{\text {Z}})$, respectively. The results assume that the intrinsic width of the ${\text {H}} ^{\pm }$ (${\text {H}} ^{\pm \pm }$) boson is $\lesssim 0.05m_{{\text {H}} ^{\pm }}$ (0.05$m_{{\text {H}} ^{\pm \pm }}$), which is below the experimental resolution in the phase space considered. The results are also interpreted in the context of the GM model including the simultaneous contributions of the ${\text {H}} ^{\pm }$ and ${\text {H}} ^{\pm \pm }$ bosons. The predicted cross sections of the ${\text {H}} ^{\pm }$ and ${\text {H}} ^{\pm \pm }$ bosons at NNLO accuracy in the GM model [21] are used for given GM parameter values of $s_{{\text {H}}}$ and $m_{{\text {H}} _{5}}$. The excluded $s_{{\text {H}}}$ values as a function of $m_{{\text {H}} _{5}}$ are shown in Fig. 8 (lower). The blue shaded region shows the parameter space for which the ${\text {H}} _{5}$ total width exceeds 10% of $m({\text {H}} _{5})$, where the model is not applicable because of perturbativity and vacuum stability requirements [21]. For the probed parameter space and $m_{\text {T}} ^{\text {V} \text {V}}$ distribution used for signal extraction, the varying width as a function of $s_{{\text {H}}}$ is assumed to have negligible effect on the result. The observed limit excludes $s_{{\text {H}}}$ values greater than 0.20–0.35 for the $m_{{\text {H}} _{5}}$ range from 200 to $1500\,{\text {GeV}}$. The limit improves the sensitivity of the previous CMS results at $13\,{\text {TeV}}$, where $s_{{\text {H}}}$ values greater than about 0.4 and 0.5 are excluded using the leptonic decay mode of the $\sigma _{\mathrm {VBF}}({\text {H}} ^{\pm \pm }) \, {\mathcal {B}}({\text {H}} ^{\pm \pm }\rightarrow {\text {W}}^\pm {\text {W}}^\pm )$ [28] and $\sigma _{\mathrm {VBF}}({\text {H}} ^{\pm }) \, {\mathcal {B}}({\text {H}} ^{\pm }\rightarrow {\text {W}}{\text {Z}})$ [29] processes, respectively, for the $m_{{\text {H}} _{5}}$ range from 200 to $1000\,{\text {GeV}}$. Tabulated results are available in the HepData database [82].

## Summary

A search for charged Higgs bosons produced in vector boson fusion processes and decaying into vector bosons, using proton–proton collisions at $\sqrt{s}=13\,{\text {TeV}}$ at the LHC, is reported. The data sample corresponds to an integrated luminosity of 137$\,{\text {fb}}^{-1}$, collected with the CMS detector between 2016 and 2018. The search is performed in the leptonic decay modes ${\text {W}}^\pm {\text {W}}^\pm \rightarrow \ell ^\pm {\upnu }\ell '^\pm {\upnu }$ and ${\text {W}}^\pm {\text {Z}}\rightarrow \ell ^\pm {\upnu }\ell '^\pm \ell '^\mp$, where $\ell , \ell ' = {\text {e}}$, ${\upmu }$. The ${\text {W}}^\pm {\text {W}}^\pm$ and ${\text {W}}{\text {Z}}$ channels are simultaneously studied by performing a binned maximum-likelihood fit using the transverse mass $m_{\text {T}}$ and dijet invariant mass $m_{\mathrm {j}\mathrm {j}}$ distributions. No excess of events with respect to the standard model background predictions is observed. Model independent upper limits at 95% confidence level are reported on the product of the cross section and branching fraction for vector boson fusion production of charged Higgs bosons decaying into vector bosons as a function of mass from 200 to $3000\,{\text {GeV}}$. The results are interpreted in the Georgi–Machacek (GM) model for which the most stringent limits to date are derived. The observed 95% confidence level limits exclude GM $s_{{\text {H}}}$ parameter values greater than 0.20–0.35 for the mass range from 200 to $1500\,{\text {GeV}}$.

## Data Availability

This manuscript has no associated data or the data will not be deposited. [Authors’ comment: Release and preservation of data used by the CMS Collaboration as the basis for publications is guided by the CMS policy as written in its document “CMS data preservation, re-use and open access policy” (https://cms-docdb.cern.ch/cgi-bin/PublicDocDB/RetrieveFile?docid=6032filename=CMSDataPolicyV1.2.pdfversion=2).]
